# A scalable empathic-mindset intervention reduces group disparities in school suspensions

**DOI:** 10.1126/sciadv.abj0691

**Published:** 2022-03-23

**Authors:** Jason A. Okonofua, J. Parker Goyer, Constance A. Lindsay, Johnetta Haugabrook, Gregory M. Walton

**Affiliations:** 1 University of California, Berkeley, Berkeley, CA, USA.; 2 Stanford University, Stanford, CA, USA.; 3 University of North Carolina, Chapel Hill, NC, USA.; 4 Multi-Tiered System of Supports, Largo, FL, USA.

## Abstract

Suspensions remove students from the learning environment at high rates throughout the United States. Policy and theory highlight social groups that face disproportionately high suspension rates—racial-minoritized students, students with a prior suspension, and students with disabilities. We used an active placebo-controlled, longitudinal field experiment (*N*_teachers_ = 66, *N*_students_ = 5822) to test a scalable “empathic-mindset” intervention, a 45- to 70-min online exercise to refocus middle school teachers on understanding and valuing the perspectives of students and on sustaining positive relationships even when students misbehave. In preregistered analyses, this exercise reduced suspension rates especially for Black and Hispanic students, cutting the racial disparity over the school year from 10.6 to 5.9 percentage points, a 45% reduction. Significant reductions were also observed for other groups of concern. Moreover, reductions persisted through the next year when students interacted with different teachers, suggesting that empathic treatment with even one teacher in a critical period can improve students’ trajectories through school.

## Teaser

An “empathic-mindset” intervention with teachers reduces suspensions and suspension disparities.

## INTRODUCTION

The widespread systemic exclusion of children from school in the United States through suspensions (*1*–*3*) is not only associated with poor academic performance and negative school and friendship experiences (*4*, *5*) but also predicts longer-run negative outcomes. For students, suspensions are associated with school dropout (*6*) and, in turn, lower earning potential (*7*) and greater substance use (*8*), crime (*9*), and risk of incarceration (*10*). Moreover, high rates of suspensions pose a fundamental threat to equal opportunity, as racial disparities in suspensions have been observed consistently for decades, as early as when schools were first desegregated (*11*). The latest national data find that Black students, for instance, are nearly four times more likely to be suspended than white students (*3*). They are also at greater risk of suffering negative life outcomes as a consequence of those suspensions (*12*).

High rates of suspension are also a problem for teachers and society at large. For teachers, difficulty managing conflict in class can undermine their ability to promote student learning (*13*). It can also be disheartening and can contribute to stress, burnout, low job satisfaction, and, ultimately, attrition from the profession (*14*, *15*). Such attrition is a national concern with declines in teacher supply in school districts across the United States (*16*). Society at large bears the costs of increased health problems, substance abuse, and crime. In economic terms, suspensions recorded for a single cohort of 10th-grade students in California and Florida produced an estimated 67,000 dropouts in the United States who, over the course of their adult lives, were estimated to generate more than $35 billion in additional government costs, including increased expenditures on health and social services and reduced tax revenue (*17*).

What causes high rates of suspensions, especially for students of color? Growing evidence points to racial bias. For instance, laboratory experiments find that teachers are more likely to knit together a series of misbehaviors as a pattern, to view a student who misbehaves as a troublemaker, and to punish them more severely, if the student is Black as compared with white (*18*). Moreover, correlational research finds an association between county aggregates of anti-Black/pro-white implicit and explicit bias and racial disparities in in- and out-of-school suspensions (*19*). Counties with more anti-Black/pro-white bias have larger disparities in suspension rates between Black and white students. Quasi-experimental evidence from North Carolina demonstrates a significant relationship between having a same-race teacher and reduced exclusionary discipline (e.g., suspensions) for Black students in particular (*20*). This reduction is driven mainly by fewer referrals for interpersonal offenses that require the subjective judgment of the teacher, such as willful defiance, pointing to a role for racial bias (*21*).

In general, strategies to contend with racial bias have aimed to reduce this bias, which, in theory, would mitigate disparities in suspension rates. While bias reduction approaches have not yet been tested in the context of school discipline, their effectiveness elsewhere has been notably limited. Typical and theory-based approaches show weak and short-lived effects at best (*22*, *23*). Moreover, reductions in anti-Black bias have not predicted changes in behaviors or outcomes (*24*).

In school, approaches to racial disparities in discipline have focused on prescriptive policies or intensive skill-building programs, each with mixed results. First, some states have banned common interpersonal offenses for which racial disparities are largest (e.g., “willful defiance”) as a basis for suspensions (*25*–*27*). These approaches may reduce this classification of offenses, but they do not necessarily prevent the offenses or exclusion from the classroom by other means (e.g., in-school suspensions). As a result, racial disparities in exclusionary discipline can remain. Second, many districts have adopted positive behavioral intervention supports (PBIS), which uses multitier models that call for heavy-touch (i.e., requiring significant resources or effort) skill-building programs such as professional “behavioral coaches” and individualized trainings for students to learn better behavior (*28*, *29*). PBIS has reduced overall suspension rates in elementary schools but is rarely effective at higher grade levels when suspension rates and racial disparities both spike (*30*). Other efforts to reduce suspensions have primarily benefited white students and have thus increased racial disparities over time (*31*). This lack of consistent effectiveness in mitigating racial disparities may be due to a lack of direct focus on the psychological mechanisms by which race-ethnicity affects teacher-student interactions and relationships.

Our theoretical approach foregrounds teacher-student interactions and bias that arises in the course of these interactions in contributing to high and disproportionate suspension rates. Even as racial stereotypes can lead teachers to be more likely to view racially stigmatized students’ misbehaviors as fixed aspects of their character (*18*), awareness of the same stereotypes can lead Black students to fear and expect unfair treatment and thus to react to any perceived bias or to disengage in ways that can be interpreted as misbehavior (*32*). These perspectives between teachers and students interact and contribute to a recursive cycle of growing mistrust and disrespect, which ultimately gives rise to large racial disparities in suspensions between racial minoritized and white students (*33*).

To effectively address this cycle, we do not seek to reduce bias in teachers. Instead, we seek to sideline bias, to make bias less controlling of teachers’ behavior. To do so, we elevate an ideal self in teachers, one that prioritizes strong working relationships with students, especially when they misbehave, and a goal—to help students grow and improve—for which bias would be maladaptive (*34*, *35*). In theory, improving teachers’ treatment of students can lead students to feel more respected by teachers when they misbehave and, in turn, to feel more respect for their teachers, especially when conflict arises, and more motivated to behave well in class (*36*).

This approach complements and extends prior research with teachers. One study found that middle and high school teachers (*N*_teachers_ = 82 for *N*_students_ = 979) who participated in My Teacher Partner (MTP), a year-long intensive and individualized training program designed to help teachers reflect on their approaches to and relationships with students, reduced racial disparities in teachers’ office referrals as compared with a randomized control group (*37*). This program, however, involved a heavy touch including multiple components, reducing its potential for scalability and scientists’ ability to isolate the causal effect. In one component, for instance, teachers watched videos of their own classrooms with trained coaches throughout the year (*38*). The study also did not examine school suspensions. Nonetheless, this research suggests that teachers’ mindsets (beliefs or attributions) about their relationships with their students are connected to their discipline practices and, moreover, that shifts in those mindsets can reduce racial disparities in disciplinary citations.

Most directly relevant, in another study, a 45- to 70-min online empathic-mindset intervention, which invited middle school teachers to describe how they carry out an ideal model for interacting with students when they misbehave, cut year-long suspension rates by 4.8 percentage points in a sample of 1682 predominantly Hispanic students (*36*). This approach used stories from teachers and students to highlight how teachers value and work to understand students’ perspectives and to sustain positive relationships during circumstances of misbehavior. In turn, participating teachers reflected on how they take this approach in their practice. However, it was tested with only a modest sample of teachers (e.g., just 31 teachers were randomized to treatment or control conditions), and the student sample was not diverse (e.g., 65% Hispanic; just 7% white and 2% Black students), so its potential to mitigate racial disparities has yet to be determined. Yet, it suggests that a brief psychological intervention with teachers can mitigate actual student suspension rates.

The current research addresses four questions. First, it provides a critical replication of the empathic-discipline intervention with a far larger and more diverse student sample (*n*_teachers_ = 66; *n*_students_ = 5822); this is the largest test to date of any randomized intervention with teachers aimed at reducing suspension rates. The potential for a brief, online, and low-cost approach to reduce suspensions is significant for theory, practice, and policy. It is essential to test the replicability of the effect in another region of the country and school district and with a different teacher and student sample.

Second, the larger and more diverse student sample allows us to evaluate whether the empathic-discipline intervention is most effective for racial minoritized students and can thus reduce racial disparities in school suspensions.

Third, the current research explores more deeply the processes by which the empathic-discipline intervention reduces student suspensions. These processes, moreover, have implications for the duration of the effect as students move from grade to grade. While both MTP and the empathic-discipline intervention are randomized to teachers with students and other teachers unaware of this condition assignment, our theory understands suspensions as emanating not from the simple judgments or behavior of one teacher alone but from patterns of interaction that develop between teachers and students, including trust or mistrust that students form about teachers in general and that teachers reflect back to students, and thus cycles of behavior that improve or worsen over time (*33*). From this perspective, a given teacher is just one avenue into a system of relationships between students and teachers, and other avenues should also be possible. Addressing students’ trust of teachers and feelings of belonging in school can also reduce disciplinary citations among racial minoritized youth (*39*). In one study, a brief intervention aimed at bolstering students’ sense of belonging at the beginning of middle school reduced disciplinary citations among Black boys over 7 years, through the end of high school. It seemed to achieve this long-term reduction by preventing the development of adverse patterns of teacher-student interaction in sixth and seventh grades and, in turn, supporting a stronger sense of belonging in school and reduced fears about being stereotyped over time (*32*).

Does the empathic-discipline intervention similarly shift students’ behaviors and, thus, improve their outcomes even beyond the treated teacher? The previous trial provides initial evidence for this theorizing. Specifically, even as only students’ math teachers were randomized to treatment or control conditions, suspensions could result from incidents that occurred at any time or place throughout the school (e.g., science class, hallway, recess, school bus) in interaction with or as observed by any staff member. In the one school in the prior trial that tracked the source of the suspension (*n* = 559 middle school students), only 7.4% of referrals were from teachers randomized to treatment or control conditions (*36*); even entirely eliminating these referrals from analyses, the treatment reduced students’ risk of suspension. The results imply that more positive treatment from one teacher triggered by the empathic-discipline intervention caused a broader improvement in students’ behavior throughout the school and school day. Here, we ask: Might this process also be developmental, a shift in fundamental ways students interact at school, and thus extend into the next academic year, even after regular contact with the treated teacher ends (*32*)? With the larger sample and by including data from the next school year, we are able to address this question.

Last, the large and diverse sample allows for exploration of the intervention’s efficacy with other policy-relevant groups, in particular students with a history of suspensions and students with a disability (i.e., special education status). Recent national policy highlights elevated suspension rates for both groups (*3*), and both may be subject to similar adverse recursive teacher-student dynamics that, we theorize, give rise to high rates of suspension. Teachers may become more punitive toward students with a history of misbehavior (*18*, *33*), expect more misbehavior, and experience greater emotional exhaustion and burnout (*40*). They may also be more concerned that students with disabilities could disrupt their classes and want them removed, as compared with typically developing students (*41*). Empathy is harder in general across group lines or when another person’s behavior or status is seen as more difficult or distressing (*42*, *43*). In these contexts, an intentional exercise that encourages teachers to take an empathic stance toward students when they misbehave may help.

We also explored effects by gender, as boys are more likely than girls to get suspended (*3*). However, boys are not broadly stigmatized in school or less likely to receive empathy and thus may not show greater reductions in suspensions with treatment.

An additional contribution of this paper is the availability of school-level administrative data that allow for the exploration of effects for students in the same schools but who did not have a teacher assigned to either condition. Thus, complementing experimental analyses, we include quasi-experimental analyses using these students as a second, nonrandomized comparison group, increasing statistical power and testing the robustness of the effects. The district discipline records also include information on the type of suspension students receive: in-school suspension (student is prohibited from going to class) or out-of-school suspension (student is prohibited from coming on campus). In-school suspensions tend to be for offenses that are more minor and more subjective and open to interpretation (e.g., willful defiance). Out-of-school suspensions tend to be for major and objective offenses (e.g., possession of a firearm). In addition, the district in which this research was conducted prohibits assigning more than 10 days of out-of-school suspension to any student in an academic semester and closely monitors out-of-school suspensions for nonviolent infractions specific to defiance and classroom disruption, limiting their use (there is no limit on in-school suspensions). For these reasons, at least in this context, by encouraging a more empathic response for misbehaviors that are open to interpretation, the empathic-discipline intervention may have more robust effects on in-school than out-of-school suspensions (*34*).

### Current research

The present research tested whether the empathic-mindset intervention could (i) reduce suspension rates in a large (*n*_students_ = 5822) and diverse (16.5% Black; 14.7% Hispanic; 57.8% white; 6.2% Asian; 0.003% American Indian; 4.5% two or more races; 49.4% female; average age, 13.05; SD = 0.66) student sample (see tables S1 to S4), (ii) mitigate racial disparities in suspension rates, and (iii) produce reductions that persist through a subsequent year. Following past research (*36*), math teachers (*n* = 66; *n*_control_ = 36, *n*_treatment_ = 30) were randomized to treatment or control conditions early in the school year. They were predominantly white (control = 84.8%; treatment = 85.7%) and female (control = 75.8%; treatment = 82.1%), as is the case nationally (80 and 77%, respectively) (*33*), and experienced (*M*_control_ = 11.42 years teaching in the district, SD = 9.39; *M*_treatment_ = 10.57, SD = 7.38) (tables S5 to S8). We focus on middle school for two reasons. First, this is when suspension rates and disparities in suspensions spike for students (*44*). Second, the intervention content focuses specifically on this context and developmental stage, including students’ experiences of early adolescence, puberty, identity development, and learning to navigate relationships with multiple adults in how it represents conflict and effective responses to misbehavior. Teachers and students were disbursed across 20 middle schools in 17 cities (table S9), all in the same large public district in the southeastern region of the United States. The schools thus had similar curricular offerings and discipline policies. By contrast, the previous evaluation of the empathic-discipline intervention was conducted in five schools in three small districts in California.

There were no significant differences in race-ethnicity, gender, age, years teaching, percentage of students suspended the year before intervention, or childhood socioeconomic status between teachers assigned to treatment (*N* = 30) versus control (*N* = 36) in semester 1, all *P*s ≥ 0.08, indicating successful randomization. There also were no treatment versus control differences in their students’ gender, previous-year suspension status (yes, no), intervention-year special education status, or probability of being American Indian, two or more races, or Asian versus white, all *P*s ≥ 0.22. However, the probability of being Black or Hispanic versus white was somewhat higher in the control condition, *P*_C_ = 32.3% versus *P*_T_ = 29.8%, *b* = 0.11, *P* = 0.047, odds ratio (OR) = 1.12, and student age was slightly lower, *M*_C_ = 13.03, *M*_T_ = 13.08, *b* = −0.047, *P* = 0.007 (Supplementary Materials; fig. S1 and tables S3 to S7).

Students were seventh- and eighth-grade students, nearly all of whom (*N* = 5533; 95%) had school record discipline data from the district from middle school (sixth and seventh grade) in the prior year. As preregistered, sixth-grade students were excluded because they did not have prior middle school suspension records (both the nature of discipline records and rates of discipline incidents differ meaningfully in elementary school); moreover, fifth-grade suspension records were not available, a limitation of the present research. Among all students who had a fall semester math teacher assigned to the treatment or control condition that same semester (*N* = 5822), 5600 (96.2%) had a single math teacher assigned to a treatment or control condition.

Specific preregistered hypotheses are indicated below in section headers (*45*). Complete model details are provided in the Supplementary Materials (p. 1). The preregistration prioritized the main effect of condition and the student race-ethnicity × condition interaction. The scope of the research expanded after initial analyses, and additional effects were explored. Which analyses were not preregistered are also marked in Results. Additional survey measures administered before teachers engaged with randomized materials are not the focus of this paper and thus not reported.

A total of 175 math teachers met eligibility for participation in the implementation period, fall semester 2017. Of these, 66 were randomly assigned to treatment or control conditions (“assigned teachers”). The remaining 109 teachers did not participate in the study during the preregistered period (i.e., the fall semester) and thus were not assigned to a treatment or control condition (“unassigned teachers”). Our final condition classifications reflected three criteria: (i) Teachers had to have been assigned to a treatment or control condition during the fall semester to be considered assigned to a condition, (ii) students had to have an assigned teacher in the fall semester, and (iii) students were classified in the treatment condition if they had at least one teacher assigned to the treatment condition during fall semester. The full sample became 173 teachers. For further detail on condition definitions, see the Supplementary Materials (p. 3). The main effect was tested with both the full sample (all teachers eligible for participation) and only teachers randomized to treatment or control conditions (excluding unassigned teachers). The former increases statistical power and provides a second, nonrandomized control group; the latter provides a pure experimental test. In all models, to reduce error variance and increase the precision of the experimental test, we control for race-ethnicity, gender, previous-year suspension status for each student, previous-year suspension rate for each math course, and previous-year suspension rate for students of each teacher. We also use missing value indicators to include students missing student, course, or teacher previous-year suspension status. For details, see the Supplementary Materials (pp. 5–8).

## RESULTS

### Main effect (preregistered)

To preserve statistical power, we first tested the main effect of condition with the full sample of teachers eligible for participation (*N* = 173) and their students (*N* = 13,210). Students with a teacher randomized to the treatment were less likely to be suspended over the course of the academic year (*M*_T_ = 17.3%), both as compared with students whose math teacher was randomized to the active control condition (*M*_C_ = 20.4%), *b* = 0.031, 95% confidence interval (CI) (−0.000 to 0.061), SE = 0.016, *z* = 1.95, *P* = 0.051 (one-tailed: *P* = 0.025), and as compared to otherwise similar students with unassigned teachers, (*M*_U_ = 22.6%), *b* = 0.053, 95% CI (0.025 to 0.080), SE = 0.014, *z* = 3.76, *P* < 0.001 (one-tailed: *P* < 0.001). The effect trended in the same hypothesized direction with the smaller sample including only those students with assigned teacher(s) (*N*_teachers_ = 66; *N*_students_ = 5,822), *b* = 0.024, 95% CI (−0.007 to 0.055), SE = 0.016, *z* = 1.51, *P* = 0.130 (one-tailed: *P* = 0.065). For brevity and clarity, the remaining results focus solely on the sample of students with at least one fall semester math teacher assigned to treatment or control conditions. Results for the model including the nonrandomized sample are described in the Supplementary Materials (tables S10 and S11).

We also distinguished two types of suspension. Among students of assigned teachers with available suspension records in the year before the intervention (*N* = 5533), 11.6% of students spent at least 1 day on in-school suspension, while 6.9% spent at least 1 day on out-of-school suspension. During the intervention year, students with a teacher randomized to the treatment were significantly less likely to receive an in-school suspension over the course of the academic year (*M*_T_ = 9.4%), as compared with students with a teacher(s) randomized to the active control condition (*M*_C_ = 14.2%), *b* = 0.048, 95% CI (0.019 to 0.076), SE = 0.014, *z* = 3.29, *P* = 0.001. The condition effect on out-of-school suspensions was not significant (*M*_T_ = 9.3%; *M*_C_ = 10.1%), *b* = 0.008, 95% CI (−0.018 to 0.033), SE = 0.013, *z* = 0.59, *P* = 0.553.

### Students’ race-ethnicity (preregistered)

**Fig. 1.**
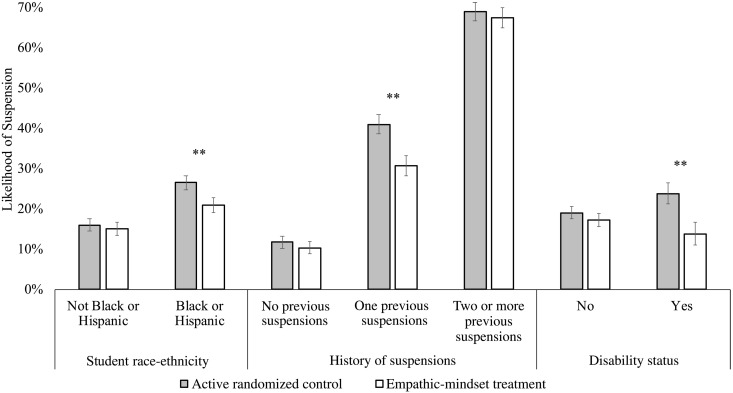
Intervention-year effects. Interaction between condition (empathic-mindset treatment versus active randomized control assignment among students’ grade 7 or 8 middle school math teachers) and (i) students’ race-ethnicity (*n*_teachers_ = 66; *n*_students_ = 5822), (ii) history of suspension (*n*_teachers_ = 65; *n*_students_ 5533), and (iii) disability (special-education assignment) status (*n*_teachers_ = 66; *n*_students_ = 5822) on student suspension rates (probability of one or more days on suspension) during the academic year of the intervention. Differences in sample sizes are due to missing data for the relevant predictor. Students who are not Black or Hispanic are white, Asian, American Indian, or students who reported two or more races. Error bars reflect ±1 SE. The significance of simple effects is denoted by **P ≤ 0.005.

Was the intervention particularly effective for students from racial groups that face negative behavioral stereotypes? Yes. As detailed in amendments to the preregistration, we coded race-ethnicity to focus on Black and Hispanic students versus any other students because (i) these groups are highlighted for national intervention efforts, as they tend to experience the highest rates of suspensions (*3*), and (ii) they had the highest suspension rates in the previous year among all racial-ethnic groups in the present sample (figs. S2 and S3; for analyses of race-ethnicity coded differently and previous-year suspension rates, see the Supplementary Materials, tables S12 to S18). In this group, the empathic-mindset treatment reduced the suspension rate by 5.6 percentage points (*M*_T_ = 20.8% versus *M*_C_ = 26.5%), *b* = 0.056, 95% CI (0.017 to 0.096), SE = 0.020, *z* = 2.79, *P* = 0.005. The suspension rate did not differ among students of other race-ethnicities by condition (*M*_T_ = 14.9% versus *M*_C_ = 15.9%), *b* = 0.009, *P* = 0.584. Black and Hispanic students had a 10.6 percentage point greater probability of suspensions than other students in the control condition, *b* = 0.106, 95% CI (0.082 to 0.130), SE = 0.012, *z* = 8.64, *P* < 0.001. This was reduced to a 5.9 percentage point disparity in the treatment condition. The race-ethnicity × condition interaction was significant, *b* = 0.047, 95% CI (0.011 to 0.083), SE = 0.019, *z* = 2.55, *P* = 0.011, reflecting a 45% reduction in the racial disparity with treatment (Fig. 1).

We also explored condition effects among Black and Hispanic students separately. The reduction was significant and similar in size for Black students, *b* = 0.057, 95% CI (0.007 to 0.106), SE = 0.025, *z* = 2.23, *P* = 0.025, and Hispanic students, *b* = 0.054, 95% CI (0.005 to 0.103), SE = 0.025, *z* = 2.14, *P* = 0.032, although the probability of suspension was higher for Black students than for Hispanic students (*M*_Black,C_ = 36.4% versus *M*_Hispanic,C_ = 16.7%; *M*_Black_,_T_ = 30.7% versus *M*_Hispanic,T_ = 11.4%).

### Students’ gender (not preregistered)

We also examined the role of gender. Neither the gender × condition interaction, *b* = 0.012, 95% CI (−0.019 to 0.043), SE = 0.016, *z* = 0.75, *P* = 0.454, nor the race-ethnicity × gender × condition interaction was significant, *b* = −0.001, 95% CI (−0.067 to 0.066), SE = 0.034, *z* = −0.02, *P* = 0.988, showing that the intervention was not differentially effective for either boys or girls (see tables S19 to S21).

### Additional student subgroups

Next, we explored intervention effects for students with a history of suspensions and for students with disabilities. There was some overlap between student race-ethnicity and these subgroups, as Black and Hispanic students were overrepresented among students with a history of suspensions and among students with disabilities (see the Supplementary Materials, tables S1 and S2). However, the following models control for student race-ethnicity (and also for gender, as in the primary models).

#### *Prior suspensions (not preregistered)*

Was the intervention effective for students with history of suspension? Yes. Students with one or more suspensions the previous year were less likely to be suspended if they had a teacher randomized to the treatment condition as compared with the control condition (*M*_T_ = 49.6% versus *M*_C_ = 56.1%), *b* = 0.065, 95% CI (0.017 to 0.113), SE = 0.024, *z* = 2.67, *P* = 0.008. Among students with no prior suspensions, the intervention-year probability of suspension did not differ significantly by condition (*M*_T_ = 10.6% versus *M*_C_ = 12.1%), *b* = 0.016, *P* = 0.352. In the control condition, students with a prior suspension had 44 percentage point higher probability of suspension relative to previously suspended students, *b* = 0.440, 95% CI (0.409 to 0.471), SE = 0.016, *z* = 27.46, *P* < 0.001. This was reduced to a 39 percentage point gap in the treatment condition. The prior suspension × condition interaction was significant, *b* = 0.050, 95% CI (0.005 to 0.095), SE = 0.023, *z* = 2.20, *P* = 0.028.

Further exploratory analyses found that the intervention was notably effective for students with one prior suspension (*M*_T_ = 30.7% versus *M*_C_ = 41.0%), *b* = 0.103, 95% CI (0.041 to 0.164), SE = 0.031, *z* = 3.27, *P* = 0.001, but not significantly effective for students with two or more prior suspensions (*M*_T_ = 67.5% versus *M*_C_ = 69.1%), *b* = 0.016, *P* = 0.59 (Fig. 1 and tables S22 to S25).

In further exploratory analyses, we found that the race × condition interaction held when also controlling for the prior suspension × condition interaction, and remained significant, *b* = 0.045, *P* = 0.017. In the same model, the prior suspension × condition interaction was somewhat reduced and became marginally significant, *b* = 0.042, *P* = 0.071 (table S26).

#### *Students with special education status (not preregistered)*

Was the intervention effective for students with special education status? Yes. These students were less likely to be suspended if they had a teacher randomized to the treatment condition (13.7%), as compared with the control condition (23.7%), *b* = 0.100, 95% CI (0.032 to 0.168), SE = 0.035, *z* = 2.87, *P* = 0.004. Students without special education status did not show a significant reduction in the treatment versus control condition (*M*_T_ = 17.0% versus *M*_C_ = 18.9%), *b* = 0.019, 95% CI (−0.012 to 0.049), SE = 0.016, *z* = 1.18, *P* = 0.238. In the control condition, students with special education status had a higher (4.8 percentage points) probability of suspension than students with this status, *b* = 0.048, 95% CI (0.002 to 0.094), SE = 0.023, *z* = 2.06, *P* = 0.039. This reversed in the treatment condition (−3.3 percentage points). The special education status × condition interaction was significant, *b* = 0.082, 95% CI (0.016 to 0.147), SE = 0.033, *z* = 2.45, *P* = 0.014 (Fig. 1 and tables S27 and S28).

### Subsequent-year suspensions for students (not preregistered)

If a student’s math teacher was randomized to the treatment condition, was the student less likely to receive a suspension the next academic year, as they interacted with new teachers? Yes.

To test this question, we examined those students who could be tracked at district middle schools through the school year following the year of randomization, *N* = 2712 students (47% of the intervention-year sample). These were students of 56 different intervention-year math teachers randomized to treatment or control conditions (85% of originally assigned teachers). Nearly all (99%) of these students were seventh graders in the intervention year and eighth graders the next year. Almost all (94%) did not have the same math teacher the next year as the teacher randomized to a treatment or control condition during the intervention year. Thus, the small percentage who did are unlikely to drive the condition effects. However, we retained all students with available data and eligible condition exposure (*N* = 2712 students) to preserve power and the integrity of random assignment.

**Fig. 2.**
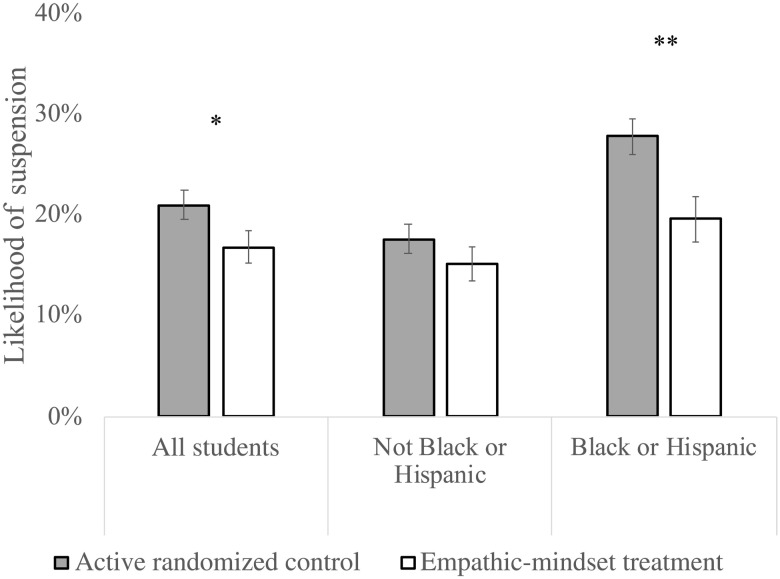
Subsequent-year effects. Condition effects (active randomized control versus empathic-mindset treatment) on suspension rates (one or more suspension days) in the year after teachers had been randomized to treatment or control conditions for all students, neither Black or Hispanic students, and Black or Hispanic students. Data reflect the subset of students who could be tracked for 2 years (*n* = 56 teachers, *n* = 2712 students). Error bars reflect ±1 SE. *P ≤ 0.050; **P ≤ 0.005.

During the intervention year, this subsample showed a slightly larger (TE_SUB_ = 3.5 percentage points versus TE_FULL_ = 2.4 percentage points) treatment versus control reduction in the probability of suspension as the full sample (*N* = 5822 students), *b* = 0.035, 95% CI (−0.005 to 0.075), *P* = 0.085. Notably, this reduction persisted through the next academic year and grew slightly (*M*_T_ = 16.7% versus *M*_C_ = 20.9%), *b* = 0.042, 95% CI (0.002 to 0.081), SE = 0.020, *z* = 2.06, *P* = 0.040 (see Fig. 2 and table S29).

We also explored the role of race-ethnicity in the longitudinal subsample. As in the full sample, intervention-year treatment effects were greater in the longitudinal subsample for Black and Hispanic students (7.6 percentage points, *P* = 0.006) than for other students (1.9 percentage points, *P* = 0.380), as reflected by a significant race-ethnicity × condition interaction, *b* = 0.057, 95% CI (0.004 to 0.111), SE = 0.027, *z* = 2.11, *P* = 0.035. In the subsequent year, the simple effects of condition for these racial-ethnic groups persisted (Black and Hispanic: 8.2 percentage points, *P* = 0.003; other: 2.5 percentage points, *P* = 0.242), as did the race-ethnicity × condition interaction, *b* = 0.057, 95% CI (0.002 to 0.112), SE = 0.028, *z* = 2.03, *P* = 0.042 (see tables S30 and 31), reflecting a 56% reduction in the racial disparity.

### Subsequent-year suspensions for teachers (not preregistered)

We also examined the rates of suspension among the new cohort of students in the subsequent year who were taught by teachers who had been assigned to the treatment or control condition in the intervention year. A total of 4915 students (*n* = 49 teachers) were eligible for this analysis as students who were enrolled in district middle schools in the subsequent year (2018–2019) as seventh (*N* = 2400) or eighth graders (*N* = 2515) and had a math teacher in 2018–2019 who had been randomized to the treatment or control condition in the fall semester of the previous school year. However, because, as in primary analyses, we sought to control for suspension status in the year before the focal outcome year, we further excluded 2915 students who were enrolled in district middle schools in the intervention year of 2017–2018 and had a math teacher randomized to the treatment or control condition that year (i.e., students with assigned math teachers in the math courses they took in both 2017–2018 and 2018–2019). For these students, prior-year suspension rates would not be a pure baseline covariate unaffected by intervention condition. As in primary (2017–2018) analyses, we also excluded students who were sixth graders in 2018–2019 from the teacher-level subsequent-year analysis. These students do not have prior suspension outcomes in middle school and nor were such records available from elementary schools. As a result of these sample limitations, the teacher-level subsequent-year analysis (*n* = 48 teachers, *n* = 2000 students) is somewhat less powered than the student-level subsequent-year analysis. When we examined probability of suspension among students with teachers assigned to treatment or control conditions the previous year, we found no effect of previous-year treatment (*M*_T_ = 20.2% versus *M*_C_ = 19.7%), *b* = −0.005, *P* = 0.754 (table S32). Additional analyses similar to the primary analyses (e.g., previous-year teacher condition by student race-ethnicity interaction) are included in the Supplementary Materials (tables S33 and S34). These reveal a nonsignificant trend for a reduction with treatment in the likelihood of suspension for Black and Hispanic students, *b* = 0.04, *P* = 0.12. This should be interpreted with caution given the increased strain on statistical power needed for these tests.

## DISCUSSION

The present research shows that a brief empathic-mindset intervention can meaningfully reduce suspension rates and mitigate racial-ethnic disparities in suspensions, replicating and extending past research. The student-wide reduction in suspensions of 2.4 percentage points [intent to treat (ITT) sample] is somewhat smaller than the 5.2 percentage point reduction in previous research, but that sample was smaller and primarily Hispanic students (*36*). The reduction is similar for strictly Black or Hispanic students, a reduction of 5.6 percentage points in the current research and 6.0 percentage points in previous research.

The results implicate a complex process of teachers’ interactions with students, and students’ consequent behavior, in students’ risk of suspensions. While teachers were the participants in the study and the subject of randomization, the focus was on their mindsets about interacting with and maintaining positive relationships with students when they misbehave, and the outcome—suspensions—was a student outcome. Moreover, this outcome reflected students’ behavior throughout the school environment and even into the next year, not just interactions with the one teacher randomized to the treatment or control condition. The findings thus point to the importance of interactions and relationships with teachers for students’ developing risk of suspensions, relative to the behavior, skills, or biases of either teachers or students alone. Moreover, they isolate teachers’ mindsets as an opportunity to unlock better outcomes above and beyond other factors.

We preregistered our hypothesis that the reduction in suspension rates would be greatest for Black and Hispanic students, giving greater confidence in this effect. This finding is important both for theory and for application. It suggests that predominately white teachers’ ability to empathize and maintain positive relationships with students in times of misbehavior—the focus of the intervention—may be most challenged with racial-minoritized students. We have theorized that this challenge is made more severe by a context that includes racial stereotypes about misbehavior for both teachers and students (*33*). Thus, an intentional focus on empathy and sustaining positive relationships can have particular benefits for minoritized students.

Two additional findings regarding students’ race-ethnicity are noteworthy. First, while the intervention reduced suspension rates for Black and Hispanic students, there was no reduction for students of other ethnicities, primarily white students, who may be less likely to face bias or experience threat because of their race or ethnicity in classrooms. Second, while Black and Hispanic students vary in many ways, including in the specific nature of the behavioral stereotypes they face (*18*, *46*), both experience identity threat in adolescence that gives rise to systems of interactions that produce discipline problems, and both can benefit from targeted exercises to reduce this threat (*32*). The present findings suggest that a more empathic mindset in teachers might level the playing field for students from both groups, supporting their development in classrooms and beyond.

In mitigating racial inequality in suspension rates, the intervention addresses a major policy priority (*3*). The implications for policy are deepened in two ways. First, this reduction was achieved in a 45- to 70-min online exercise with teachers. It was not necessary, for instance, to reach students individually (*32*, *39*) or to implement an intensive professional development program for teachers (*37*, *38*). Most participating teachers completed only the initial 45-min session, suggesting that the single session was impactful. The intervention could be brief precisely because it did not attempt to build teachers’ skills or to systematically change their practices. It focused only on mindsets. While we believe there are important nuances in the effective delivery of the empathic-mindset intervention (e.g., that the exercise is presented to teachers as honorific, not remedial) (*47*), and while there are surely important boundary conditions around its effects (*48*), the empathic-discipline intervention is certainly easier and cheaper for schools and districts to administer than alternatives. An important direction for future research is to rigorously identify these boundary conditions, and thus where the intervention can be used effectively (*49*). More broadly, the results illustrate the potential to target the mindsets of “gatekeepers”—people in positions of power—to improve the experiences of many (*50*). Here, a shift in dozens of teachers’ mindsets improved outcomes for thousands of students.

Second, even as the empathic-mindset intervention benefitted Black and Hispanic students most, the direction of the effect was positive for all groups and significant for other policy-relevant groups susceptible to biased perception and a lack of empathy, namely, students who had been suspended previously and those with disabilities (*40*, *41*). Yet, it is notable that the intervention was not differentially effective for boys as compared with girls, even as boys are more likely to get suspended than girls (*3*). Previous research suggests that boys tend to engage in a broader range of misbehaviors than girls, including offenses that might be less malleable to intervention (i.e., drugs and weapons) (*21*). Future research may further explore how teachers respond to these misbehaviors and whether there are other opportunities to reduce the elevated risk of suspensions boys face.

The results also provide further insight into for whom and how the intervention works. First, it is notable that the empathic-mindset intervention focused on teachers’ approach to relationships with students in general when behavioral issues arise. It did not name or provide individualized guidance for relationships with any particular student or students. This quality may inform its efficacy and its limitations. Even as the treatment was highly effective in reducing suspensions for students with one prior suspension (8.3% of students with available data, who showed a 10.3 percentage point reduction), consistent with its goal in preventing the escalation of disciplinary responses following misbehavior (*9*, *10*, *23*), it did not significantly reduce suspensions for students for whom this escalation process had not yet begun, students who had no suspension the previous year (84.3% of students with available data, who showed a 1.3 percentage point reduction). It also did not reduce suspensions for students for whom this process was better established, students who had two or more prior suspensions (8.7% of students with available data, who showed a 1.6 percentage point reduction). Students with one prior suspension may represent a “sweet spot” for the intervention. Laboratory experimentation has shown that the intervention’s content can reduce the degree to which teachers view a student with a prior misbehavior as a troublemaker and the severity with which they would punish this student (*34*). Students with a more extensive history of misbehavior may require a more individuated approach that focuses on the educator’s specific relationship with that student (*36*).

The focus on students in general may similarly inform the differential effect of the treatment on reducing in-school, as compared with out-of-school, suspensions. While both kinds of suspensions remove students from the learning environment, in-school suspensions are often for less severe offenses, for more directly relational offenses (e.g., “insubordination”), and for offenses that entail greater subjective judgment. This relative ambiguity may provide more room for a shift in teachers’ mindset and an improvement in patterns of interaction to reduce removals from the learning environment. Out-of-school suspensions, by contrast, typically require documented rationale and remanding of the student to the custody of a legal guardian. They may require a more individuated approach that focuses on particular patterns of previous misbehaviors (e.g., multiple in-school suspensions) and more severe and objective offense (e.g., fighting), and may require involvement of other actors (e.g., principals) (*51*). As noted, the present district also prohibits assigning more than 10 days of out-of-school suspension to any student in an academic semester and discourages and closely monitors out-of-school suspensions for nonviolent infractions specific to defiance and classroom disruption. While such policies may reduce out-of-school suspensions (*34*), this policy does not apply to in-school suspensions. The present research demonstrates the benefits of the empathic-discipline intervention above and beyond this policy, at least in reducing in-school suspensions. Yet, this context also raises questions about how variable state and district policy contexts interact with interventions that focus on the mindsets of educators as they contend with and respond to misbehavior (*34*, *48*). For instance, in districts or states that have not yet implemented such policies (*52*), would the empathic-mindset intervention reduce out-of-school suspensions as well?

Second, it was notable that the reduction in suspensions for students persisted into the next academic year. To persist even as students interact with new teachers, it seems likely that the treatment shifted adolescents’ developing beliefs about the kinds of relationships they have or can have with teachers, beliefs that underlie their behavior in school (*32*). Perhaps, the intervention’s strategic focus helps teachers empathize with students during key interactions and offer adolescents what they crave (e.g., respect). Previous laboratory experimentation has found that students feel greater respect for teachers and motivation to behave well in class when a teacher has responded in an empathic manner to their misbehavior (*36*). Also consistent with this theorizing, past direct-to-student interventions, when delivered at developmentally important junctures, can have powerful, lasting benefits for adolescents at least in part, it seems, by sustaining their trust of teachers and sense of belonging in school (*32*, *37*, *53*). A limitation of the present study is that we did not assess such student beliefs directly. Understanding how empathic treatment from a single teacher contributes to this process is an important direction for future research.

The present research also explored whether the effect would persist for teachers into the next year, as they interact with new students. There was not strong evidence for persistence at the teacher level, although limitations of statistical power constrain this analysis (there was a nonsignificant trend for Black and Hispanic students). However, it may also be important that experienced teachers, unlike middle school students, are not developing new beliefs about the kinds of interactions and relationships they can have in general with students; such beliefs have already been formed. Instead, teachers are developing new beliefs about specific cohorts of students. If so, persistent benefits at the teacher level may require reactivation of empathic-mindset themes and their application to each new cohort of students. Future research may further explore this question, including barriers to persistent benefits at the teacher level from one school year to the next and how to overcome these.

It will also be exciting to explore effects in adjacent age groups, both among sixth-grade students, ideally in K-8 schools that have the same context, policies, and procedures for discipline in elementary and middle school, and among high school students.

The empathic-discipline intervention invites us to ask: What could be possible if all (middle school) teachers were to take part in the intervention? Could we see suspension rates drop at large, especially for the most at-risk students? Would classrooms be under greater control, with teachers empowered to create better learning environments? Yet, to get there, important questions and limitations of the present research arise, especially involving uptake among teachers and heterogeneity at the teacher and school/school district level. First, in the present research, participation in the empathic-mindset exercise was brief and accessible to teachers (and free and promoted by their district), yet most eligible teachers did not participate. What factors contribute to teachers’ decisions to participate or not in a professional development opportunity like this? How can we increase participation? In the present study, participating teachers tended to be female, older in age, have more years of teaching experience, less likely to be teaching remedial courses, and to have students with lower average suspension rates in the year before intervention (table S8). Might other teachers experience different motivation or hurdles to participation? Second, if many teachers participate, with what kinds of teachers would the intervention be more effective, or less? Would some teachers not see a way to put it into practice in their context? Would it be redundant and therefore not impactful for others? Third, if the intervention were distributed more widely, how would students’ experience shift if several of their teachers, rather than just one, received the intervention? Fourth, this study was conducted in a single large and diverse school district, replicating and extending findings elsewhere. If the intervention was delivered with a nationally representative sample of districts and with representative teachers within districts, in what kinds of districts and with what kinds of teachers would the intervention be most effective? What are the contextual affordances, both structural and psychological, necessary for the intervention to reduce suspension rates (*48*)? Such questions are essential for understanding the generalizability of the intervention benefits and thus inform both theory and policy.

With these open questions in mind, the results highlight the importance of empathy in pivotal teacher-student relationships and its capacity to benefit children at the most risk of disparate treatment.

## MATERIALS AND METHODS

### Empathic-mindset: A focus on teacher-student relationships

The “empathic-mindset” intervention is a brief online reading and reflection exercise. It invites teachers to reflect on the pivotal opportunity they have to help students grow and learn when they misbehave, including to listen to students and understand their perspective, and to sustain positive relationships in circumstances of misbehavior. It uses targeted articles, narratives, and written reflection exercises to represent this approach to misbehavior as normative, ideal, and intuitive, as common wisdom backed by research. The materials address students’ social and emotional development, teachers’ capacity to help students develop prosocial skills, and the value of prioritizing students’ perspectives, especially in times of conflict or when they misbehave. They thus encourage an empathic approach to understanding and responding to student misbehavior. Participating teachers are treated as experts in interacting with students when they misbehave and invited to share their expertise in response to the intervention materials to support future, younger teachers.

The first page of the module introduces teachers to the program:

“In this web module, we will share with you some… research. Then, we will ask you for your input as a professional educator. We are especially interested in your thoughts about how teachers like you can and do use these ideas to have better interactions with students and to improve their lives” (*36*).

Thus, teachers are told that their responses will be incorporated in trainings for future teachers and treating teachers as exemplary and as experts rather than as problematic or as recipients of an intervention. Teachers then read how “everyone has a personal story about a great teacher who influenced his or her life”, and these stories often involve the teacher effectively communicating that they care for and support students, including by listening to and valuing student’s perspectives. The materials elevate this ideal professional self by emphasizing that doing so can also help teachers interact with students in ways that nurture their growth into responsible, motivated young adults.

These ideas are reinforced in student and teacher narratives, relatable stories that illustrate how understanding students’ perspectives can be enlightening and useful. For example, a Black seventh grader shares:

In middle school, I did not feel like I belonged. It seemed like the teachers always called on the other students. So I did not pay attention in class and sometimes I got in trouble. One day I got detention and, instead of just sitting there, my teacher talked with me about what happened. He really listened to me. And then he told me that he had trouble sometimes in middle school but that it gets better. It felt good to know I had someone I could trust in school (*36*).

Throughout, these narratives are presented as paradigmatic examples, not teachers’ specific students. Their purpose is to encourage teachers to learn more about their own students and to give them tools to do so, rather than to use to substitute for doing so or to serve as a basis for assumptions about their own students.

The materials are interactive. For instance, teachers are asked how they maintain positive relationships with their own students even when they misbehave. Last, teachers are asked to reflect on the materials they had reviewed and their own experiences to write a letter to a new teacher in their district to help them navigate relationships with students. Such “saying-is-believing” tasks can help people articulate a psychological message for themselves and connect it to their own experience and thus use it going forward.

The control condition module was similar in length and exercises. However, the content addressed how technology can enhance teachers’ capacity to engage their students. While this also involve supporting students’ learning, it does not focus on responses to misbehavior (*34*).

While 96.2% of students only had one math teacher, some students had multiple math teachers. However, there was demographic balance among students with multiple math teachers in the control and treatment conditions (see tables S3 and S4). More details are included in the Supplementary Materials (p. 6).

Before the experiment began, approval from the Institutional Review Board at University of California at Berkeley was given. The consent form was the first page teachers saw when they followed a link to the study.
