# Supplementary material for: A scalable empathic-mindset intervention reduces group disparities in school suspensions
Source: Sci Adv. 2022 Mar 23;8(12):eabj0691. doi: 10.1126/sciadv.abj0691 (PMC8942350; doi:10.1126/sciadv.abj0691)
Supplement: Supplementary file 1 — Supplementary Materials and Methods Tables S1 to S37 Figs. S1 to S3 [file sciadv.abj0691_sm.pdf]

Science Advances
AAAS

Supplementary Materials for

A scalable empathic-mindset intervention reduces group disparities in school suspensions

Jason A. Okonofua*, J. Parker Goyer, Constance A. Lindsay,
Johnetta Haugabrook, Gregory M. Walton

*Corresponding author. Email: okonofua@berkeley.edu

Published 23 March 2022, *Sci. Adv.* **8**, eabj0691 (2022)
DOI: 10.1126/sciadv.abj0691

This PDF file includes:

Supplementary Materials and Methods
Tables S1 to S37
Figs. S1 to S3

Materials

Hypothesis and Analytic Plan for Main Text Analyses

1. **Pre-Registered Analyses.** We pre-registered (*34*) the following hypotheses before data analyses were performed:
    1.1. Consistent with Okonofua, Paunesku, and Walton (2016), the intervention, as compared to control condition, will reduce the probability of receiving a suspension.
    1.2. The intervention, as compared to control condition, will reduce the probability of receiving a suspension for Black, Hispanic, American Indian, and multiple race students. Further, there will be a two-way interaction between the intervention effect and the effect of student race such that the reduction in suspensions is driven by a reduction in suspensions for Black, Hispanic, American Indian, and multiple race students. Research shows that students from racially stigmatized groups face a heightened risk of stereotypes barring them from meaningful relationships with teachers (*28*). (As discussed below, we later amended the pre-registration to simplify how we categorized race-ethnicity.)
2. **Non-Pre-Registered Analytic Plan.** We also followed an analytic plan (not pre-registered) to explore other relevant outcomes:
    2.1. Is the intervention effective for students at the most risk for suspensions (*3*)?
        2.1.1. Is the intervention effective for students with disabilities (eligible for special education)?
        2.1.2. Is the intervention effective for students with a history of suspension(s) (*9-11*)?
    2.2. Does the intervention effect persist for students into the next school year, reducing reduce their suspensions as they have new teachers?
    2.3. Does the intervention effect persist for teachers into the next school year, reducing suspensions for the new students they teach?
3. **Additional pre-registered exploration**
    3.1. We will explore a three-way interaction among the intervention effect and the effects of student race and gender. The intervention effect will be larger for either (3a) Black, Hispanic, American Indian, and multiple race girls than for other race × gender groups or (3b) Black, Hispanic, American Indian, and multiple race boys than for other race × gender groups. We did not specify a direction for the gender effect because past research was consistent with both possibilities.

Methods

Procedure

Math teachers at 20 middle schools (for school characteristics, see Table S4) in a large and diverse school district were recruited to participate in the randomized placebo-controlled field experiment. Teachers were told the purpose of the modules was to collect their perspectives on various aspects of their profession. In Fall 2017, teachers were offered an opportunity to complete a 45-minute online module embedded in a Qualtrics survey. Though they were also offered an opportunity to complete a second 25-minute booster module in the Spring semester, only six teachers did so.

The intervention and control materials were identical to previous research with the empathic-discipline intervention (*22*). Both conditions included narratives, stories from other teachers and from students, and personal reflection exercises. Further, both were represented as honorific rather than as remedial—as an effort to learn from experienced teachers to help future

teachers in teacher training programs. In the treatment condition, the materials asked teachers how they valued students' perspectives and maintained high-quality relationships with students, even when they misbehave. In the control condition, the materials asked teachers how they used technological tools to keep students engaged with lessons.

District records identified 200 teachers who taught at least one math class to 6th, 7th, or 8th graders across these schools We defined math teachers as teachers who taught at least one math course with at least five students. Consequently, we excluded 481 student-math course–teacher–school rows in which the teacher–school combination occurred fewer than 5 times in the dataset. These rows may have represented courses with fewer than 5 students or may have been an irregularity in the district data. This was primarily an issue for the three-condition dataset. Only 177 such rows needed to be dropped from the ITT dataset. Our focus was on 7th and 8th grade students, students who had previous middle-school disciplinary records in middle school and for whom we could therefore control for previous-year suspension rates in middle school. 5th-grade suspension rates were unavailable, and suspension rates in general spike in middle school, rendering 5th grade records less relevant even if they had been available. Therefore, we removed students whose grade level during the intervention year was 6th grade from the final student sample (this in turn removed teachers who primarily or exclusively taught 6th grade math classes). A total of 175 teachers, who primarily taught 7th and 8th grade math, were eligible. Teachers who met eligibility criteria (e.g., taught math) were randomly assigned to condition immediately after consent. Randomization was stratified by school, that is, to try to ensure that as close to 50% of teachers from each school would be assigned to treatment versus control. Analyses examined 173 of these 175 teachers because two such teachers, who themselves were non-assigned, only taught students who had another teacher who had been assigned to condition. In an effort to preserve the integrity of the intent-to-treat sample and reduce the inclusion of multiple rows in the dataset when students had multiple math teachers, we removed data for a student's non-assigned teachers for students who had at least one assigned teacher before executing analyses. Collectively, these 173 teachers taught 13,210 7th and 8th grade students across the 20 middle schools. Of the 109 teachers who began the survey containing the intervention materials, 90 did so in the Fall implementation period as intended, and 66 reached the point of random assignment and were randomly assigned to condition (*N*=36 control; *N*=30 treatment). These 66 teachers form the intent-to-treat (ITT) sample. An additional 19 teachers (109 – 90) accessed the session 1 module only in the Spring of 2018. Because these teachers were not randomly assigned in Fall 2017, they were excluded from ITT analyses (*N*=5,822 students) and coded as "no-assignment," along with teachers who never started the module, in analyses comparing students of treatment- and control-condition teachers to students of non-assigned teachers (*N*=13,210). For the CONSORT diagram, see Figure S1.

The primary analyses featured in the manuscript text are ITT analyses, which compare suspension outcomes for students with at least one treatment-condition math teacher in Fall 2017 to students with no treatment-condition math teachers but at least one control-condition math teacher in Fall 2017. As such, these analyses exclude students who did not have a math teacher randomized to condition in the Fall of 2017. Analyses that sought to compare suspension outcomes for students of treatment-condition and control-condition teachers (*N*=66 teachers) to other math teachers at the participating schools (*N*=107 teachers) included a third "no-assignment" condition group. The non-assigned condition included the following types of students: grade-7 or 8 students who only took math courses in Spring 2018 and students whose Fall-semester math teachers did not receive a randomized condition assignment in Fall 2017.

Non-assigned teachers differed in several ways from assigned teachers (see Table S8). With the exception of the main effect of condition, all analyses focus on students of the 66 teachers who were randomized to condition in Fall 2017 (ITT analyses). There were no exclusions of teachers based on engagement with or completion of the materials.

The school district provided discipline records, course records, and demographic records for all students in all district middle schools for the year before (AY 2016-2017), during (AY 2017-2018), and after (AY 2018-2019) the intervention, including the 13,210 seventh and eighth grade students these teachers taught. However, there were occasional missing data.

Analytical Approach

All analyses were conducted in STATA Version 16.1 using multilevel linear probability random effects models (implemented by the *mixed* regression command) in which students were nested within math course title-teacher groups (e.g.., classrooms) within teachers within schools.

The goal was to understand the effect of treatment on students' year-long probability of suspension as a function of whether teachers were exposed to either treatment or control materials in semester 1, the Fall 2017 semester. A student was considered to be in the treatment condition if the student had at least one math course in Fall 2017 in which the math teacher was randomly assigned to the treatment that semester. A student was considered to be in the control condition if the student had no math courses in Fall 2017 in which the teacher was randomly assigned to the treatment in Fall 2017 and at least one math course in Fall 2017 in which the math teacher was randomly assigned to the control in Fall 2017.

In the ITT sample (N=5,822), the goal was to compare only students in randomized treatment versus control conditions. To achieve this all students without a fall-semester math course taught by a math teacher randomized to condition in the fall were excluded from analyses. In the larger sample (N=13,210; hereafter the ALL sample), where the goal was to increase power for the focal comparison and compare grade 7 and 8 students in both randomly assigned conditions to students of all other math teachers at the participating schools, these students were included as a "non-assigned" condition. Non-assigned students could include 1) students whose Fall-2017 math courses had no math teachers assigned to condition in Fall 2017 (because these teachers never started the study materials, because these teachers never reached the point of random assignment, or because they only started the materials in Spring 2018) students who had no Fall-2017 math courses (only Spring-2018 math courses) or 3) students whose first exposure to an assigned math teacher(s) was in the spring semester (only their spring semester math courses were taught by assigned math teachers). For the main effect of condition only, the manuscript text reports results obtained in both the ITT (N=5,822) and the larger sample including non-assigned students (N=13,210). All other results reported reflect the ITT sample.

The treatment condition served as the reference category in typical models (except for specific purposes, such as to compute the main effect of race in the control condition). The effect of condition was represented by a single contrast in treatment-versus-control-only models (1=Control; 0=Treatment) and by two contrasts in treatment versus control and non-assigned models (1=Control, 0=Otherwise; 1=Non-Assigned, 0=Otherwise; hence the treatment condition has a code of 0 for both indicators). All models controlled for student-level dichotomous race-ethnicity, gender, and suspension status in the previous school year (0=No, student received no days on suspension in the 2016-2017 school-year, 1=Yes, student received at least one day on suspension in the 2016-2017 school-year). Additionally, we controlled for continuous average suspension rates in the previous school year at the course-level (district wide, averaging across

schools that offered the math course and all district teachers who taught the math course) and teacher-level (averaging across all students taught by the teacher, in any math course) (see below). When students had multiple math courses (because they had multiple courses with the same teacher or multiple math teachers), we computed an average previous suspension rate across the student's courses for the course-level and teacher-level previous suspension rates, respectively. We used three separate missing value indicators to indicate whether students were missing data for previous suspensions (0 = No, 1 = Yes), one for each of the three previous suspension covariates (student-level, course-level, and teacher-level). We did not have missing data for any other covariates.

In all models (both excluding and including non-assigned students), we mean-centered course-level and teacher-level prior suspensions on the course-level mean and teacher mean, respectively, for math courses and teachers in the ITT sample so the intercept would represent the treatment-condition mean for a student in a course with average levels of previous suspensions relative to all ITT-sample math courses and a teacher with average levels of previous suspensions relative to all ITT-sample math teachers. Consistent with prior research (*35*), we mean-centered student-level prior suspensions on the mean for a student's race-ethnicity × gender group, so the intercept in the model would represent the treatment-condition mean for the average student in a given race-ethnicity × gender group, given that suspension rates differ across such groups. When they functioned primarily as a covariate in the model, as in analyses assessing the main effect of condition, the remaining dichotomous indicators (student race-ethnicity, gender, and missing value indicators) were mean-centered on the student-level mean for that variable. When the goal was to test an interaction between a specific dichotomous variable or variables (e.g., student race-ethnicity, gender, or previous suspension status) and condition, the relevant dichotomous indicator was left as a 0/1 variable and allowed to interact with each condition contrast (while all other dichotomous indicators were student-mean-centered). The analysis assessing the interaction between condition and student special education status (0=No disability; Yes=At least one disability) added a 0/1 predictor for special education status and its interaction with condition to a model in which all other student-level predictors (race-ethnicity, gender, and previous suspension status) were student-mean-centered. We computed simple effects of condition using the *margins* command. These reflect the control versus treatment difference (or non-assignment versus treatment difference) in the probability of suspension when the student group of interest was defined as 0 for the moderator and had average values for all other covariates. Thus positive coefficient values reflect lower treatment means relative to control or non-assigned means and correspond to treatment reductions.

Measures and coding methods

Dependent variable. Consistent with previous research (*23*), the primary dependent variable was whether a student received at least one suspension day (=1) versus not (=0) as a result of a discipline referral from any teacher or staff member at any time during the 2017-2018 school year, the year of intervention. For students who had school records at district schools in both 2017-2018 and 2018-2019, we also assessed whether a student received at least one suspension day (=1) versus not (=0) during the school year immediately following the intervention year (2018-2019).

Student-level condition. To accommodate the few students who had multiple assigned math teachers, we defined a student as in the treatment condition if they had at least one Fall-2017 math teacher who was randomly assigned to the empathic mindset intervention in Fall

2017. We defined a student as in the control condition if they had at least one Fall-2017 math teacher who was randomly assigned to the control condition in Fall 2017 but no Fall-2017 teachers assigned to the treatment condition that semester. The ALL sample included all other students in the "no-assignment category": these students were present in grade 7 or 8 math courses but did not have a Fall-2017 math teacher who received a random assignment to condition in Fall 2017.

Previous suspensions at the student-, course-, and teacher-level covariates. To control for previous student-level suspensions, we used a single contrast to distinguish students who had received no suspension days in the prior academic year (=0) from students who had received one or more suspension days (=1). Because this district offers a common math curriculum across schools and students in specific types of math classes may have different propensity for receiving suspensions, independent of teacher, we also controlled for the average number of suspensions associated with each math course title across schools in the prior school year. We further controlled for the average suspension rate of students in the prior school year who had the student's same math teacher during the previous school year.

Student demographic covariates. Students belonged to one of six racial-ethnic groups: White, Asian, Black, Hispanic, American Indian, Mixed (two or more races). These racial-ethnic groups differed in both proportional representation and average previous suspension rates in our sample in ways that are consistent with national data. When models simply sought to control for race-ethnicity (e.g., as when testing the main effect of condition), we used two contrasts: Black and Hispanic (=1) versus White (=0) and all other groups (=1) versus White (=0). Here Other indicates Asian, American Indian, or Students with Two or More Races. This approach most closely reflected the proportional representation of racial-ethnic groups in our sample, while also preserving some distinctions between groups in ways that partially reflected prior group discipline patterns. In the 13,210 sample, both Black and Hispanic students had similar proportional representation (16.8% and 17.6% respectively) while all other minority groups each had ≤ 5.3% representation. This was also true in the 5,822 ITT sample (16.5% Black, 14.7% Hispanic, all other minority groups ≤ 6.2% representation respectively). This coding scheme grouped together the two largest minoritized groups in our sample that tend to face disadvantage in disciplinary contexts (Black and Hispanic). Black and Hispanic students also had significantly higher suspensions the year prior to the intervention than White students (See Figure S3). The reference category in regression models with this coding scheme was White students, which was the most numerous racial-ethnic group in the sample (55.7% of the ALL sample and 57.8% of the ITT sample; See Figure S2). Since this model best reflects the nature of this particular sample, we use this racial coding as a mean-centered covariate for race-ethnicity. Gender was coded as female (=1) versus male (=0) and was also student-mean-centered.

Effects by student race-ethnicity and gender. To increase power for tests of interactions between condition and student race-ethnicity, we converted these two dichotomous race-ethnicity variables into a single race-ethnicity contrast that distinguished Black or Hispanic (=1) from not Black or Hispanic students (=0). This coding scheme was more parsimonious and required only one interaction term with condition yet still grouped the two largest minoritized groups in our sample that tend to face disadvantage in disciplinary contexts (Black and Hispanic students). We also report results based on other coding schemes (including the pre-registered Black, Hispanic, American Indian, and Two or More Races vs White or Asian) in supplemental analyses (Tables S14-15).

Effects by student suspension history. We first tested whether condition effects varied as a function of receiving one or more 2016-2017 discipline referrals that resulted in suspension day(s) (=1) versus no (=0) 2016-2017 discipline referrals that resulted in suspension days. This required a single predictor (1=One or More Prior Suspensions; 0=No Prior Suspensions) interacted with condition. Second, we disaggregated the former category into receiving exactly one discipline referral that resulted in a suspension day(s) versus receiving two or more discipline referrals that resulted in a suspension day(s). This created three categories: students with no, one, and two or more prior suspensions. We tested these as two contrasts, with no suspensions serving as the reference category and each of the other two coded as 1 (One Prior Suspension = 1, Otherwise = 0; Two or More Prior Suspensions = 1, Otherwise = 0).

Effects by student with special education status. We used a dichotomous variable for whether students had any disability based on special education status as listed in school records (0=No – Student had no disabilities; 1=Yes – Student had at least one disability). These disabilities included one or more of 17 disability statuses defined by the district, including physical, academic, social, or emotional behavioral disabilities.

Additional Analyses Tables: Subsequent year missing data (not pre-registered)

It is possible that students who were suspended during the intervention year were more likely to leave the school district. Might this have impacted subsequent year outcomes in this research? To answer this question, we focused on students who were 7th graders in the intervention year, as nearly all students included in the student-level subsequent year analysis were 7th graders (rather than 8th graders) in the intervention year. In addition, leaving the district is more ambiguous for 8th graders, as it could represent the positive outcome of being promoted to high school/grade 9. The below models show suspensions during the intervention year did predict log-odds of leaving the school district (Table S35), b=0.78 (log-odds), p<.001. In percentage-point terms, students who were suspended in the intervention year had a 5.8 percentage point higher probability of leaving the district in the subsequent year (11.2%) relative to non-suspended students (5.5%). Exploring this further by condition, this phenomenon was primarily an issue for the control condition. There was no significant difference in probability of leaving the district for suspended (M=7.6%) or non-suspended students (M=5.2%) in the treatment condition, b=0.024 (percentage points), p=.28. Yet control-condition students who were suspended (M=13.0%) were significantly more likely than control-condition students who were not suspended (M=5.7%) to leave the district the next school year, b=0.073 (percentage points), p<.001. (Table S36-S37). However, these simple effects should be interpreted with caution, as the intervention-year suspension status × condition interaction did not reach significance, b=0.51 (log-odds), p=.19.

Importantly, this selection process works *against* the subsequent-year treatment effect. If students who, in 7th grade, would otherwise have been at high risk of receiving a suspension the next year but did not because they left the district, in part as a consequence of having a 7th-grade math teacher who was assigned to the control condition, this would depress the control-condition suspension mean in the subsequent year relative to what it would have been if all students had been retained in the district into 8th grade. Thus, the treatment vs control effects we observe in the subsequent year may underestimate the true effect.

Tables

Table S1. *Proportions representing the relationships among the three moderators for probability of suspension: students' race-ethnicity, prior-year suspension status, and special-education status.*

	Proportion of Sample (N=5822)	Proportion of Students Suspended in Prior Year (N=870)	Proportion of Students in Special Education in Intervention Year (N=349)
Black	16.5%	42.8%	30.7%
Hispanic	14.7%	14.0%	16.9%
American Indian	0.3%	0.2%	0.3%
Two or More Races	4.5%	4.1%	4.3%
Asian	6.2%	2.1%	2.3%
White	57.8%	36.8%	45.6%
Total	100.0%	100.0%	100.0%

Note. The table presents the proportion of students with teachers assigned to condition in the ITT sample (N=5,822) in each racial-ethnic group, as well as the proportion of each racial-ethnic group who received at least one suspension in the pre-intervention school year (AY 2016-2017) and the proportion of each racial-ethnic group with any special education status in the intervention school-year (AY 2017-2018). In this sample, prior suspension status was available for 5,533 students (15.7% or N=870 received at least one suspension in the prior school year) and special education status was available for 5,821 students (6.0% or N=349 had one or more disabilities in the intervention year).

Table S2. *Regression coefficients representing the relationships among the three moderators for probability of suspension*

	Coef.	Std. Err.	t	p	[95% CI]	
Prior Suspensions → Black or Hispanic student	0.309	0.017	18.692	0.000	0.277	0.341
Special Education Status → Black or Hispanic student	0.174	0.025	6.816	0.000	0.124	0.224
Special Education Status → Prior Suspensions	0.218	0.021	10.505	0.000	0.177	0.259

Note. The table presents a comparison of linear regression coefficients for prior-year (AY 2016-2017) suspension status (1=Received at least one discipline referral resulting in suspension days; 0=otherwise) predicting student's race-ethnicity is Black or Hispanic, intervention-year (AY 2017-2018) special-education status predicting student's race-ethnicity is Black or Hispanic, and intervention-year (AY 2017-2018) special-education status predicting prior-year suspension status in the ITT sample (N=5,822).

Table S3. *Covariate balance between student condition in the ITT sample, overall and for one versus multiple ITT math teachers*

	Means			Control vs Treatment Contrast		
	ITT Sample	Treatment	Control	ITT Sample	One ITT Math Teacher	Multiple ITT Math Teachers
Student Race is Black/Hispanic vs White	0.312	0.298	0.323	0.024*	0.022	0.079
Student Race is Other vs White	0.109	0.106	0.111	0.005	0.005	0.009
Student Gender is Female	0.494	0.485	0.500	0.015	0.021	-0.150*
Student Suspended in Previous Year	0.157	0.160	0.156	-0.004	-0.010	0.144*
Student Has Special Education Status	0.060	0.064	0.057	-0.008	-0.011	0.070
Student Has Multiple Math Teachers	0.038	0.037	0.039	0.002	--	--
Sample Size	5822	2470	3352	5822	5600	222

Note. The table presents the balance for key covariates for students classified as being in the treatment or control condition in the ITT sample (N=5,822). In this sample, 5600 students ($n_{control}$=3,222, $n_{treatment}$=2,378) had one Fall-semester math teacher randomly assigned to condition that semester; 222 students ($n_{control}$=130, $n_{treatment}$=92) had multiple Fall-semester math teachers randomly assigned to condition that semester (though 116 or 52% of these were assigned to the same condition). Contrasts are the difference in means or proportions. Significance is based on logistic regression coefficients (all measures were dichotomous). ***$p \leq .001$, **$p \leq .01$, *$p \leq .05$.

Table S4. *For students with multiple math teacher assigned to condition (n=222; $n_{control}$=130, $n_{treatment}$=92), the relationships among the student demographics (students' race-ethnicity, gender, prior-year suspension status, special-education status, multiple teachers or not) by condition.*

	Students with Multiple ITT Math Teachers			
	Means			Contrasts
Var_Desc	ITT Sample	Treatment	Control	Control vs Treatment
Student Race is Black/Hispanic vs White	0.514	0.467	0.546	0.079
Student Race is Other vs White	0.081	0.076	0.085	0.009
Student Gender is Female	0.477	0.565	0.415	-0.150*
Student Suspended in Previous Year	0.327	0.244	0.388	0.144*
Student Has Special Education Status	0.117	0.076	0.146	0.070
Sample Size	222	92	130	

Note. Contrasts are the difference in means or proportions. Significance is based on regression coefficients (logistic for dichotomous measures). ***$p \leq .001$, **$p \leq .01$, *$p \leq .05$.

Table S5. *District-Provided Ethnicity Coded by Census Definitions, by Condition/Group, for All District Middle School Teachers*

	Math Teachers				All Teachers		
	Study Teachers			Non-Eligible Teachers			
	Treatment (N=30)	Control (N=36)	No Assignment (N=107)	Exclude (N=27)	All Math Teachers (N=200)	Non-Math Teachers (N=459)	All Teachers (N=659)
Hispanic	0.0%	2.8%	6.5%	7.4%	5.0%	4.4%	4.6%
Black	6.7%	8.3%	5.6%	11.1%	7.0%	7.4%	7.3%
White	80.0%	77.8%	60.7%	70.4%	68.0%	66.2%	66.8%
Asian	6.7%	2.8%	0.9%	7.4%	3.0%	0.9%	1.5%
American Indian	0.0%	0.0%	0.0%	0.0%	0.0%	0.4%	0.3%
Unknown	6.7%	8.3%	26.2%	3.7%	17.0%	20.7%	19.6%
All Races	100.0%	100.0%	100.0%	100.0%	100.0%	100.0%	100.0%

Note. We coded all district-provided race-ethnicities with which a teacher identified according to census definitions. We first coded teachers who identified as Hispanic for at least one ethnicity. We then coded teachers who identified as Black, not Hispanic; followed by White, not Hispanic or Black; followed by Asian, not White, Hispanic, or Black; followed by American Indian, not Black, Hispanic, White, or Asian. "Exclude" teachers taught only or primarily 6th grade classes (N=25) or were non-assigned teachers who only taught students of other assigned teachers (N=2) (see Figure S1). Percentages reflect teachers with available race-ethnicity data for any of the four academic years provided by the district, prioritizing the race-ethnicity reported in the intervention year (2017-2018) if available. The sample size below each group represents all teachers in that group, whether or not (coded unknown) they had available data.

Table S6. *Math Teacher Demographic Variables, by All Teachers, Math Teachers, and Study Math Teachers*

	Teachers in AY 2017-2018		
	Study Teachers (N=173)	All Math Teachers (N=200)	All Teachers (N=659)
Missing in District Demographic Records	19.1%	17.0%	19.6%
Missing Student-Level Discipline Data in Previous Year	26.6%	26.5%	22.6%
Census Ethnicity is White	83.6%	81.9%	83.0%
Gender is Female	67.9%	68.7%	76.2%
Age in Intervention Year	41.85	42.01	44.01
	(11.02)	(11.01)	(11.73)
Years Teaching Experience as of Intervention Year	9.14	9.34	10.19
	(7.95)	(7.89)	(8.59)
Average Probability of Suspension Among Teacher's Students in Previous Year	0.22	0.22	0.22
	(0.15)	(0.15)	(0.16)

Table S7. *Math Teacher Covariate Balance, by Randomized Condition, for Study Math Teachers*

	Group Means		Condition Difference
	Treatment (N=30)	Control (N=36)	Control vs Treatment
Census Ethnicity is White[D]	85.7%	84.8%	0.93
Gender is Female[D]	82.1%	75.8%	0.68
Age in AY 2017-2018	44.61	44.82	0.21
Years Teaching Experience in AY 2017-2018	10.57	11.42	0.85
Average Suspensions Received by Teacher's Students in Previous Year	0.18	0.14	-0.04
Total distinct math course titles taught	2.10	2.22	0.12
Taught any Grade 6 math course[D]	33.3%	25.0%	0.67
Taught any Grade 7 math course[D]	66.7%	69.4%	1.14
Taught any Grade 8 math course[D]	53.3%	52.8%	0.98
Taught any remedial math course[D]	40.0%	47.2%	1.34
Taught any regular math course[D]	46.7%	47.2%	1.02
Taught any advanced math course[D]	76.7%	86.1%	1.89

Note. The table reports the condition means for teachers randomly assigned to the treatment or control condition in Fall 2017 for demographic variables (available from district teacher records), suspension-related variables (computed from student discipline records), and grade levels and kinds of math courses taught (computed from district course records). The average suspension rate represents the average probability of suspensions received by the teacher's students (a student who had that teacher at any point during the academic year) at any time during the pre-intervention academic year (the suspension could be issued by any staff member). Linear regression (logistic regression) was used for continuous (dichotomous, indicated by superscript D) indicators. Logistic regression coefficients are represented as odds ratios, the ratio of the odds of having a value of 1 versus 0 on the indicator in the control (none) versus the treatment condition. Demographic indicators and prior suspension rates could not be computed for teachers for whom these records were unavailable. The sample size below each group represents all teachers in that group, whether or not they had available data for a given covariate. ***$p \leq .001$, **$p \leq .01$, *$p \leq .05$

Table S8. *Comparison of Assigned and Non-Assigned Study Math Teachers*

	Group Means		Condition Difference
	Assigned (N=66)	Non-Assigned (N=107)	Assigned vs Non-Assigned
Census Ethnicity is White	85.2%	82.3%	1.24
Gender is Female	78.7%	59.5%	2.51*
Age in AY 2017-2018	44.72	39.63	5.09**
Years Teaching Experience in AY 2017-2018	11.03	7.67	3.36**
Average Suspensions Received by Teacher's Students in Previous Year	0.16	0.27	-0.11***
Total distinct math course titles taught	2.17	2.17	0.00
Taught any Grade 6 math course	28.8%	34.6%	0.76
Taught any Grade 7 math course	68.2%	72.9%	0.80
Taught any Grade 8 math course	53.0%	52.3%	1.03
Taught any remedial math course	43.9%	64.5%	0.43**
Taught any regular math course	47.0%	67.3%	0.43**
Taught any advanced math course	81.8%	51.4%	4.25***

Note. This table compares assigned (teachers who were randomized to condition in Fall 2017) versus non-assigned teachers (teachers who elected not to participate in the study or who were randomized to condition in Spring 2018). Survey variables (childhood hometown, childhood SES, and psychometrics) were unavailable for the vast majority of non-assigned teachers. Specifications are otherwise the same as Table S7. ***$p \leq .001$, **$p \leq .01$, *$p \leq .05$

Table S9. *Comparison of participating schools on key indicators.*

School	Total School Enrollment	Students Suspended Previous Year (%)	Students Suspended Intervention Year (%)	Racial Composition of School (%)				
				Asian	Black	Hispanic	White	Other
1	3278	19.7%	18.4%	5.2%	14.1%	19.8%	56.1%	4.8%
2	3067	21.4%	21.5%	4.0%	11.3%	16.0%	64.2%	4.6%
3	2506	20.6%	31.3%	9.1%	18.1%	17.3%	50.8%	4.7%
4	2227	21.5%	24.5%	4.4%	13.2%	15.2%	62.0%	5.3%
5	2218	20.3%	21.2%	3.7%	16.0%	12.5%	62.9%	4.9%
6	2083	27.5%	32.0%	4.5%	38.8%	11.9%	40.2%	4.6%
7	1974	24.4%	27.2%	2.4%	14.7%	28.5%	50.0%	4.5%
8	1899	17.9%	18.0%	4.7%	13.0%	15.9%	61.5%	4.8%
9	1791	25.8%	28.5%	6.0%	21.0%	15.8%	52.7%	4.5%
10	1784	24.4%	29.8%	5.3%	20.3%	21.1%	48.0%	5.1%
11	1750	30.1%	37.1%	3.2%	34.4%	15.8%	41.6%	5.1%
12	1739	22.0%	24.3%	8.1%	15.2%	22.0%	50.2%	4.4%
13	1691	15.2%	12.5%	4.4%	9.3%	15.0%	67.7%	3.5%
14	1563	14.3%	14.1%	5.7%	27.5%	10.7%	51.3%	4.8%
15	1436	25.8%	32.9%	7.0%	20.1%	17.7%	49.6%	5.7%
16	1387	31.3%	33.2%	3.5%	41.2%	12.4%	37.7%	5.2%
17	1345	23.7%	27.3%	4.6%	18.8%	24.3%	46.6%	5.6%
18	1229	14.0%	9.2%	3.2%	9.2%	17.0%	65.9%	4.7%
19	1161	24.9%	25.8%	3.5%	12.1%	16.1%	63.5%	4.7%
20	592	12.3%	12.1%	4.5%	8.3%	10.9%	72.2%	4.1%

Note. Other consists of American Indian and multiple-race students.

Main Text Analyses Tables (pre-registered)

All models predict the probability of receiving at least one suspension day in the intervention year unless otherwise indicated. All models controlled for the following covariates (not listed in tables for simplicity): mean-centered student race-ethnicity, mean-centered student gender, whether or not the student received a suspension in the prior year (student-level), average suspension rate for students taking same math course in the prior year district-wide (course-level), average suspension rate among students who had the same math teacher the prior year (teacher-level), and missing value indicators for prior suspensions data at the student-, course-, and teacher-level. All effects tables present the intercept (the treatment-condition mean for a student in the relevant reference category) and the condition difference (control or none vs treatment) from this mean from the corresponding model. When student race-ethnicity, gender, or prior suspension status is the focal moderator, it is coded as 0/1 rather than mean-centered.

Table S10. *Key Regression Coefficients from Model of Main Effect of Control v. Treatment Effect in ITT Sample*

At Least One Suspension Day	Coef.	Std.Err.	z	p	[95% CI]	
Control v. Treatment	0.024	0.016	1.512	0.130	-0.007	0.055
Intercept (Treatment Mean)	0.168	0.016	10.270	0.000	0.136	0.200

At Least One In-School-Suspension	Coef.	Std.Err.	z	p	[95% CI]	
Control v. Treatment	0.048	0.014	3.289	0.001	0.019	0.076
Intercept (Treatment Mean)	0.094	0.014	6.898	0.000	0.067	0.121

At Least One Out-of-School Suspension	Coef.	Std.Err.	z	p	[95% CI]	
Control v. Treatment	0.008	0.013	0.593	0.553	-0.018	0.033
Intercept (Treatment Mean)	0.093	0.011	8.754	0.000	0.073	0.114

Note. *N*=5,822 students, with 66 different intervention-year teachers.

Table S11. Key Regression Coefficients from *Model of Main Effect of Control or No Assignment v. Treatment Effect in ALL Sample*

	Coef.	Std.Err.	z	p	[95% CI]	
Control v. Treatment	0.031	0.016	1.955	0.051	0.000	0.061
No Assignment v. Treatment	0.053	0.014	3.760	0.000	0.025	0.080
Intercept (Treatment Mean)	0.173	0.016	10.865	0.000	0.142	0.205

Note. *N*=13,210 students, with 173 different intervention-year teachers.

Table S12. *Key Regression Coefficients from Model testing the interaction between Control v. Treatment and whether student race-ethnicity is Black or Hispanic or not in the ITT Sample*

	Coef.	Std.Err.	z	p	[95% CI]	
Control v. Treatment	0.009	0.017	0.548	0.584	-0.024	0.042
Student Race is Black or Hispanic	0.059	0.014	4.137	0.000	0.031	0.087
Student Race × Control v. Treatment	0.047	0.019	2.549	0.011	0.011	0.083
Intercept (Non-Black-Hispanic Treatment Mean)	0.149	0.017	8.900	0.000	0.117	0.182

Note. *N*=5,822 students, with 66 different intervention-year teachers. Student race-ethnicity is a dichotomous variable defined as 1 if race-ethnicity is Black or Hispanic and 0 if race-ethnicity is not Black or Hispanic.

Table S13. Simple *Effects of Control v. Treatment from Model testing the interaction between Control v. Treatment and whether student race-ethnicity is Black or Hispanic or not in the ITT Sample*

	Coef.	Std.Err.	z	p	[95% CI]	
Not Black or Hispanic	0.009	0.017	0.548	0.584	-0.024	0.042
Black or Hispanic	0.056	0.020	2.787	0.005	0.017	0.096

Note. *N*=5,822 students, with 66 different intervention-year teachers. Simple effects were computed using the model shown in Table S12.

Table S14. Simple *Effects of Control v. Treatment from Separate Models testing the interaction between Control v. Treatment and whether student race-ethnicity is Black (Hispanic) in the ITT Sample*

	Coef.	Std.Err.	z	p	[95% CI]	
Not Black	0.016	0.016	1.037	0.300	-0.015	0.047
Black	0.057	0.025	2.235	0.025	0.007	0.106
Not Hispanic	0.016	0.016	0.971	0.332	-0.016	0.047
Hispanic	0.054	0.025	2.143	0.032	0.005	0.103

Note. *N*=5,822 students, with 66 different intervention-year teachers. In the top two rows, student race-ethnicity is a dichotomous variable defined as 1 if race-ethnicity is Black and 0 if race-ethnicity is not Black. In the bottom two rows, student race-ethnicity is a dichotomous variable defined as 1 if race-ethnicity is Hispanic and 0 if race-ethnicity is not Hispanic.

Table S15. *Key Regression Coefficients from Model testing the interaction between Control v. Treatment and whether student race-ethnicity is Black, Hispanic, or Other (American Indian or Two or More Races) or not in the ITT Sample*

	Coef.	Std.Err.	z	p	[95% CI]	
Control v. Treatment	0.010	0.017	0.560	0.575	-0.024	0.043
Student Race is Black or Hispanic or Other	0.069	0.014	5.032	0.000	0.042	0.096
Student Race × Control v. Treatment	0.039	0.018	2.163	0.031	0.004	0.074
Intercept (White or Asian Treatment Mean)	0.143	0.017	8.423	0.000	0.110	0.176

Note. *N*=5,822 students, with 66 different intervention-year teachers. Student race-ethnicity is a dichotomous variable defined as 1 if race-ethnicity is Black or Hispanic or American Indian or Two or More Races and 0 if race-ethnicity is White or Asian.

Table S16. *Key Regression Coefficients from Model testing the interaction between Control v. Treatment and whether student race is Black or not in the ITT Sample*

	Coef.	Std.Err.	z	p	[95% CI]	
Control v. Treatment	0.016	0.016	1.037	0.300	-0.015	0.047
Student Race is Black	0.167	0.019	8.937	0.000	0.130	0.204
Student Race × Control v. Treatment	0.040	0.024	1.666	0.096	-0.007	0.088
Intercept (Non-Black Treatment Mean)	0.140	0.017	8.454	0.000	0.108	0.173

Note. *N*=5,822 students, with 66 different intervention-year teachers. Student race is a dichotomous variable defined as 1 if race is Black and 0 if race is non-Black. The simple effects of condition for this model are presented in the top two rows in Table S14.

Table S17. *Key Regression Coefficients from Model testing the interaction between Control v. Treatment and whether student race is Black or White in the ITT Sample*

	Coef.	Std.Err.	z	p	[95% CI]	
Control v. Treatment	0.006	0.017	0.357	0.721	-0.027	0.039
Student Race is Black v. White	0.157	0.019	8.131	0.000	0.119	0.195
Student Race is Other v. White	-0.028	0.015	-1.897	0.058	-0.057	0.001
Race (B v. W) × Control v. Treatment	0.051	0.025	2.043	0.041	0.002	0.100
Race (O v. W) × Control v. Treatment	0.034	0.019	1.741	0.082	-0.004	0.071
Intercept (Non-Black Treatment Mean)	0.149	0.017	8.630	0.000	0.115	0.182

Note. *N*=5,822 students, with 66 different intervention-year teachers. Student race is two dichotomous variables defined as 1 if race is Black and 0 if race is White and as 1 if race is Other and 0 if race is White.

Table S18. *Key Regression Coefficients from Model testing the interaction between Control v. Treatment and whether student race is Hispanic or White in the ITT Sample*

	Coef.	Std.Err.	z	p	[95% CI]	
Control v. Treatment	0.006	0.017	0.369	0.712	-0.027	0.040
Student Race is Hispanic v. White	-0.040	0.018	-2.175	0.030	-0.075	-0.004
Student Race is Other v. White	0.087	0.015	5.664	0.000	0.057	0.117
Race (H v. W) × Control v. Treatment	0.048	0.024	2.037	0.042	0.002	0.095
Race (O v. W) × Control v. Treatment	0.032	0.020	1.578	0.115	-0.008	0.071
Intercept (Non-Black Treatment Mean)	0.152	0.017	8.939	0.000	0.118	0.185

Note. *N*=5,822 students, with 66 different intervention-year teachers. Student race is two dichotomous variables defined as 1 if race is Hispanic and 0 if race is White and as 1 if race is Other and 0 if race is White.

Additional Gender Analyses Tables

Table S19. *Key Regression Coefficients from Model testing the interaction between Control v. Treatment and whether student gender is Female or not in the ITT Sample (non-preregistered)*

	Coef.	Std.Err.	z	p	[95% CI]	
Control v. Treatment	0.018	0.018	1.020	0.308	-0.017	0.053
Student Gender is Female	-0.094	0.012	-7.828	0.000	-0.118	-0.071
Student Gender × Control v. Treatment	0.012	0.016	0.749	0.454	-0.019	0.043
Intercept (Male Mean)	0.215	0.017	12.295	0.000	0.181	0.249

Note. N=5,822 students, with 66 different intervention-year teachers. Student gender is a dichotomous variable defined as 1 if gender is Female and 0 if gender is male.

Table S20. *Key Regression Coefficients from Model testing the interactions among Control v. Treatment and whether student race-ethnicity is Black or Hispanic or not and whether student gender is Female or not in the ITT Sample (pre-registered).*

	Coef.	Std.Err.	z	p	[95% CI]	
Control v. Treatment	0.004	0.019	0.217	0.829	-0.034	0.042
Student Race is Black or Hispanic	0.035	0.019	1.855	0.064	-0.002	0.072
Student Gender is Female	-0.110	0.015	-7.527	0.000	-0.138	-0.081
Student Race × Gender	0.049	0.026	1.911	0.056	-0.001	0.100
Student Race × Control v. Treatment	0.047	0.025	1.908	0.056	-0.001	0.096
Student Gender x Control v. Treatment	0.011	0.019	0.581	0.561	-0.027	0.049
Student Race × Gender × Control v. Treatment	-0.001	0.034	-0.015	0.988	-0.067	0.066
Intercept (Not Black or Hispanic Male Mean)	0.203	0.018	11.108	0.000	0.167	0.239

Note. N=5,822 students, with 66 different intervention-year teachers. Student race-ethnicity is a dichotomous variable defined as 1 if race-ethnicity is Black or Hispanic and 0 if race-ethnicity is not Black or Hispanic. Student gender is a dichotomous variable defined as 1 if gender is Female and 0 if gender is male.

Table S21. *Simple effects of condition from Model testing the interactions among Control v. Treatment and whether student race-ethnicity is Black or Hispanic or not and whether student gender is Female or not in the ITT Sample*

	Coef.	Std.Err.	z	p	[95% CI]	
Not Black or Hispanic Males	0.004	0.019	0.217	0.829	-0.034	0.042
Black or Hispanic Males	0.052	0.024	2.109	0.035	0.004	0.099
Not Black or Hispanic Females	0.016	0.020	0.794	0.427	-0.023	0.054
Black or Hispanic Females	0.062	0.025	2.498	0.012	0.013	0.111

Note. N=5,822 students, with 66 different intervention-year teachers. Simple effects were computed using the model shown in Table S20.

Main Text Analyses Tables: Primary outcomes (not pre-registered)

Table S22. *Key Regression Coefficients from Model testing the interaction between Control v. Treatment and dichotomous student history of suspension in the ITT sample*

	Coef.	Std.Err.	z	p	[95% CI]	
Control v. Treatment	0.015	0.016	0.930	0.352	-0.017	0.047
Student Was Previously Suspended	0.390	0.017	22.752	0.000	0.356	0.423
Student Suspension History × Control v. Treatment	0.050	0.023	2.195	0.028	0.005	0.095
Intercept (Mean for Students with Zero Prior Suspensions)	0.106	0.016	6.523	0.000	0.074	0.138

Note. N=5,533 students, with 65 different intervention-year teachers. Student history of suspension is defined as 1 (v. 0) if student received one or more (versus zero) discipline referrals in the previous year (AY 2016-2017) resulting in suspension days, for students in the ITT Sample with available data.

Table S23. *Simple effects of condition from Model testing the interaction between Control v. Treatment and dichotomous student history of suspension in the ITT sample*

	Coef.	Std.Err.	z	p	[95% CI]	
No Suspensions in Previous Year	0.015	0.016	0.930	0.352	-0.017	0.047
One or More Suspensions in Previous Year	0.065	0.024	2.669	0.008	0.017	0.113

Note. N=5,533 students, with 65 different intervention-year teachers. Simple effects were computed using the model shown in Table S22.

Table S24. *Key Regression Coefficients from Model testing the interaction between Control v. Treatment and two types of suspension history*

	Coef.	Std.Err.	z	p	[95% CI]	
Control v. Treatment	0.013	0.015	0.893	0.372	-0.016	0.043
Prior Suspension (1 v. 0)	0.204	0.022	9.092	0.000	0.160	0.248
Prior Suspension (2 or more v. 0)	0.572	0.022	26.122	0.000	0.529	0.615
Prior Suspension (1 v. 0) × Control v. Treatment	0.089	0.031	2.924	0.003	0.029	0.149
Prior Suspension (2 v. 0) × Control v. Treatment	0.003	0.029	0.088	0.930	-0.054	0.059
Intercept (Mean for Students with Zero Prior Suspensions)	0.103	0.015	6.735	0.000	0.073	0.133

Note. N=5,533 students, with 65 different intervention-year teachers. Student history of suspension is defined with two dichotomous variables (1=Student received one discipline referral in previous year resulting in suspension days; 0=Otherwise; 1=Student received two or more discipline referrals in previous year resulting in suspension days; 0=Otherwise), for students in the ITT Sample with available data. Students receiving zero suspension days in the previous year serve as the reference category.

Table S25. *Simple Effects of Condition from Model testing the interaction between Control v. Treatment and two types of suspension history*

	Coef.	Std.Err.	z	p	[95% CI]	
No Suspensions in Previous Year	0.013	0.015	0.893	0.372	-0.016	0.043
One Suspension in Previous Year	0.103	0.031	3.269	0.001	0.041	0.164
Two or More Suspensions in Previous Year	0.016	0.030	0.537	0.591	-0.042	0.074

Note. N=5,533 students, with 65 different intervention-year teachers. Simple effects were computed using the model shown in Table S24.

Table S26. *Key Regression Coefficients from Model testing the interactions among Control v. Treatment, student race, student history of suspension*

	Coef.	Std.Err.	z	p	[95% CI]	
Control v. Treatment	0.003	0.017	0.149	0.881	-0.031	0.036
Student Race is Black or Hispanic	-0.010	0.015	-0.713	0.476	-0.039	0.018
Student Suspension History	0.394	0.017	22.894	0.000	0.361	0.428
Student Race × Control v. Treatment	0.045	0.019	2.381	0.017	0.008	0.083
Student Suspension History × Control v. Treatment	0.042	0.023	1.803	0.071	-0.004	0.087
Intercept (Not Black or Hispanic Non-Suspended Mean)	0.107	0.017	6.411	0.000	0.074	0.140

Table S27. Key Regression Coefficients from *Model testing the interaction between Control v. Treatment and student special education status*

	Coef.	Std.Err.	z	p	[95% CI]	
Control v. Treatment	0.019	0.016	1.179	0.238	-0.012	0.049
Student Has Disability Status	-0.034	0.024	-1.388	0.165	-0.081	0.014
Student Disability Status × Control v. Treatment	0.082	0.033	2.452	0.014	0.016	0.147
Intercept (Mean for Students with No Disabilities)	0.170	0.016	10.477	0.000	0.138	0.202

Note. N=5,822 students, with 66 different intervention-year teachers. Student special education status was defined as 1 if a student had any disability in the intervention year (AY 2017-2018) and 0 otherwise.

Table S28. *Simple effects of condition* from *Model testing the interaction between Control v. Treatment and student special education status*

	Coef.	Std.Err.	z	p	[95% CI]	
No Disability Status	0.019	0.016	1.179	0.238	-0.012	0.049
Has Disability Status	0.100	0.035	2.872	0.004	0.032	0.168

Note. N=5,822 students, with 66 different intervention-year teachers. Simple effects were computed using the model shown in Table S27.

Main Text Analyses Tables: Subsequent year outcomes (not pre-registered)

Table S29. *Key Regression Coefficients from Model of Main Effect of Control v. Treatment among students who could be tracked in district schools for two years.*

Intervention Academic Year (AY 2017-2018)	Coef.	Std.Err.	z	p	[95% CI]	
Control v. Treatment	0.035	0.020	1.724	0.085	-0.005	0.075
Intercept (Treatment Mean)	0.168	0.020	8.281	0.000	0.128	0.208

Subsequent Academic Year (AY 2018-2019)	Coef.	Std.Err.	z	p	[95% CI]	
Control v. Treatment	0.042	0.020	2.056	0.040	0.002	0.081
Intercept (Treatment Mean)	0.167	0.016	10.280	0.000	0.135	0.199

Note. N=2,712 students, with 56 different intervention-year teachers. The model was fit for students with district school records in the intervention year and subsequent academic year, classified according to their 7th grade math teacher(s)' condition assignment during the intervention year. Nearly all such students were 7th graders in the intervention year and 8th graders in the subsequent year. The model excluded students with suspension records for both years who were not exposed to an assigned math teacher in Fall 2017.

Table S30. *Key Regression Coefficients from Model testing the interaction between Control v. Treatment and student race-ethnicity is Black or Hispanic for students who could be tracked in district schools for two years.*

Intervention Academic Year (AY 2017-2018)	Coef.	Std.Err.	z	p	[95% CI]	
Control v. Treatment	0.019	0.021	0.878	0.380	-0.023	0.061
Student Race is Black or Hispanic	0.046	0.021	2.165	0.030	0.004	0.088
Student Race × Control v. Treatment	0.057	0.027	2.108	0.035	0.004	0.111
Intercept (Non-Black-Hispanic Treatment Mean)	0.151	0.021	7.282	0.000	0.111	0.192

Subsequent Academic Year (AY 2018-2019)	Coef.	Std.Err.	z	p	[95% CI]	
Control v. Treatment	0.025	0.021	1.171	0.241	-0.017	0.066
Student Race is Black or Hispanic	0.046	0.022	2.073	0.038	0.002	0.089
Student Race × Control v. Treatment	0.057	0.028	2.031	0.042	0.002	0.112
Intercept (Non-Black-Hispanic Treatment Mean)	0.151	0.017	8.957	0.000	0.118	0.184

Note. *N*=2,712 students, with 56 different intervention-year teachers. Student race-ethnicity is a dichotomous variable defined as 1 if race-ethnicity is Black or Hispanic and 0 if race-ethnicity is not Black or Hispanic. The model was fit for students with district school records in the intervention year and subsequent academic year, classified according to their 7th grade math teacher(s)' condition assignment during the intervention year. Nearly all such students were 7th graders in the intervention year and 8th graders in the subsequent year. The model excluded students with suspension records for both years who were not exposed to an assigned math teacher in Fall 2017.

Table S31. *Effects of treatment by student race-ethnicity (Black or Hispanic (=1) from Not Black or Hispanic (=0) on intervention year and subsequent year suspension rate for teachers.*

Intervention Academic Year (AY 2017-2018)	Coef.	Std.Err.	z	p	[95% CI]	
Not Black or Hispanic	0.019	0.021	0.878	0.380	-0.023	0.061
Black or Hispanic	0.076	0.028	2.736	0.006	0.022	0.131

Subsequent Academic Year (AY 2018-2019)	Coef.	Std.Err.	z	p	[95% CI]	
Not Black or Hispanic	0.025	0.021	1.171	0.241	-0.017	0.066
Black or Hispanic	0.082	0.028	2.929	0.003	0.027	0.137

Note. *N*=2,712 students, with 56 different intervention-year teachers. Simple effects were computed using the models shown in Table S30.

Table S32. *Key Regression Coefficients from Model of Main Effect of Control v. Treatment of suspensions the subsequent year for new students of teachers assigned to condition during the intervention year.*

Subsequent Academic Year	Coef.	Std.Err.	z	p	[95% CI]	
Control v. Treatment	-0.005	0.016	-0.313	0.754	-0.037	0.027
Intercept (Treatment Mean)	0.202	0.013	15.861	0.000	0.177	0.227

Table S33. *Key Regression Coefficients from Model of Interaction between Control v. Treatment and student race-ethnicity of suspensions the subsequent year for new students of teachers assigned to condition during the intervention year.*

Subsequent Academic Year	Coef.	Std.Err.	z	p	[95% CI]	
Control v. Treatment	-0.031	0.020	-1.528	0.126	-0.070	0.009
Student Race is Black or Hispanic	0.102	0.024	4.240	0.000	0.055	0.149
Student Race × Control v. Treatment	0.072	0.032	2.260	0.024	0.010	0.134
Intercept (Non-Black-Hispanic Treatment Mean)	0.165	0.015	10.696	0.000	0.135	0.195

Note. *N*=2,000 students, with 48 different intervention-year teachers. Student race-ethnicity is a dichotomous variable defined as 1 if race-ethnicity is Black or Hispanic and 0 if race-ethnicity is not Black or Hispanic. Students who had an assigned teacher in 2017-2018 were excluded from this analysis.

Table S34. *Effects of treatment by student race-ethnicity (Black or Hispanic (=1) from Not Black or Hispanic (=0) on subsequent year suspension rate for new students of teachers assigned to condition during the intervention year.*

Subsequent Academic Year	Coef.	Std.Err.	z	p	[95% CI]	
Not Black or Hispanic	-0.031	0.020	-1.528	0.126	-0.070	0.009
Black or Hispanic	0.041	0.027	1.540	0.124	-0.011	0.093

Note. *N*=2,000 students, with 48 different intervention-year teachers. Simple effects were computed using the models shown in Table S33. Students who had an assigned teacher in 2017-2018 were excluded from this analysis.

Additional Analyses Tables: Subsequent year missing data (not pre-registered)

Table S35. *Key Log-odds Coefficients from Model of Main Effect of 7th grade intervention year suspensions on missing discipline records in subsequent year*

	Coef.	Std.Err.	z	p	[95% CI]	
Suspended (0 v. 1 or more)	0.781	0.170	4.595	0.000	0.448	1.114
Intercept	-2.849	0.090	-31.820	0.000	-3.024	-2.673

Table S36. *Key Log-odds Coefficients from Model testing the interaction between 7th grade intervention year suspensions and Control v. Treatment on missing discipline records in subsequent year for students.*

	Coef.	Std.Err.	z	p	[95% CI]	
Control v. Treatment	0.403	0.331	1.216	0.224	-0.246	1.052
Suspended (0 v. 1 or more)	0.091	0.182	0.502	0.616	-0.265	0.448
Suspended × Control v. Treatment	0.507	0.388	1.305	0.192	-0.254	1.268
Intercept	-2.902	0.140	-20.760	0.000	-3.176	-2.628

Table S37. *Simple effects of condition from Model testing the interaction between 7th grade intervention year suspensions and Control v. Treatment on missing discipline records in subsequent year for students.*

	Coef.	Std.Err.	z	p	[95% CI]	
Condition effect for students not suspended in intervention year	0.005	0.009	0.505	0.614	-0.014	0.023
Condition effect for students suspended in intervention year	0.054	0.028	1.919	0.055	-0.001	0.109
Suspension effect for control students	0.024	0.022	1.077	0.282	-0.020	0.067
Suspension effect for treatment students	0.073	0.020	3.714	0.000	0.035	0.112

Note. Simple effects were computed using the model shown in Table S20.

Figures

Figure S1. *Teacher Inclusion Criteria and Condition Assignments*

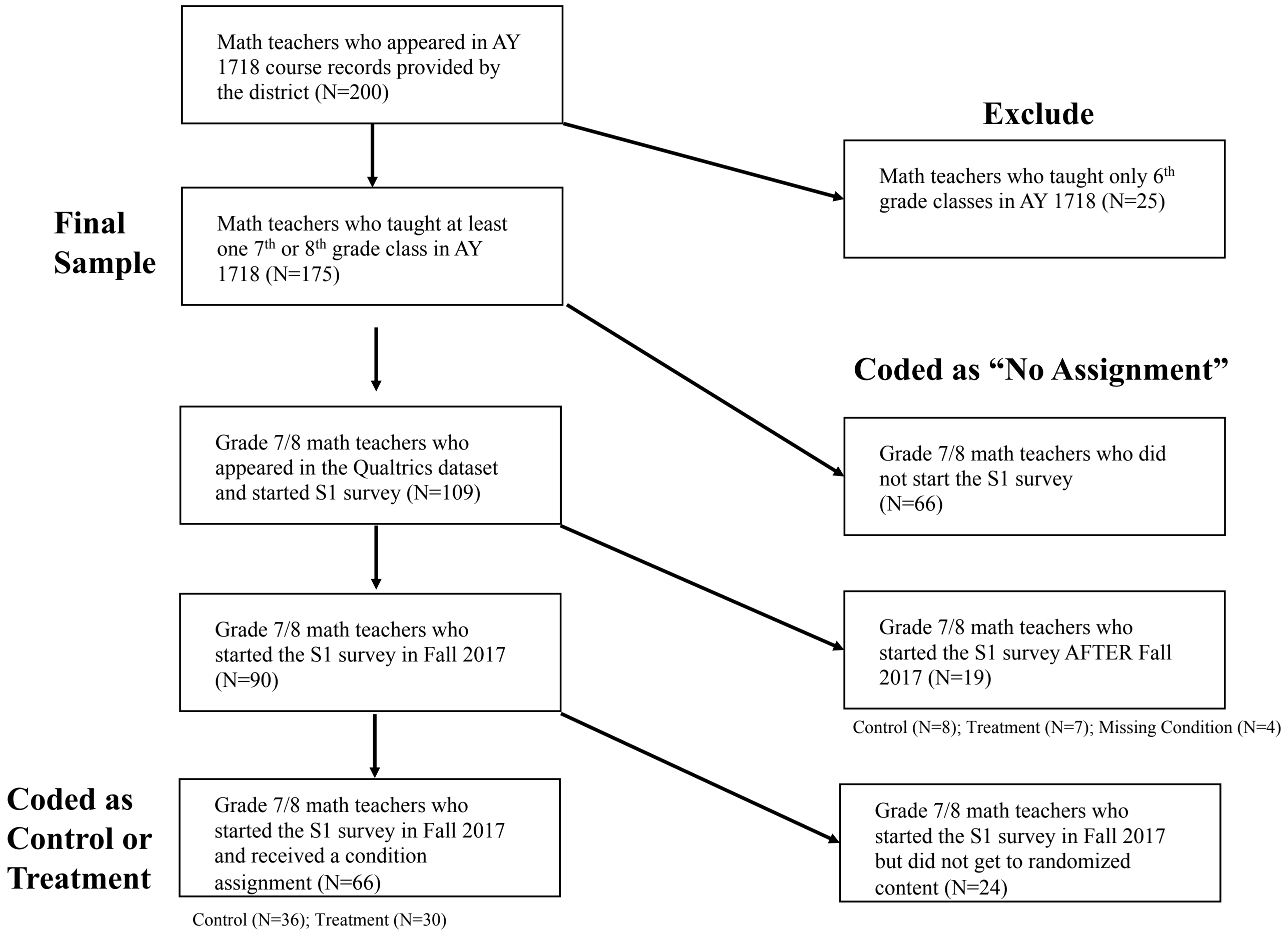

Figure S2. *Proportion of Students by Race-Ethnicity During Intervention Year*

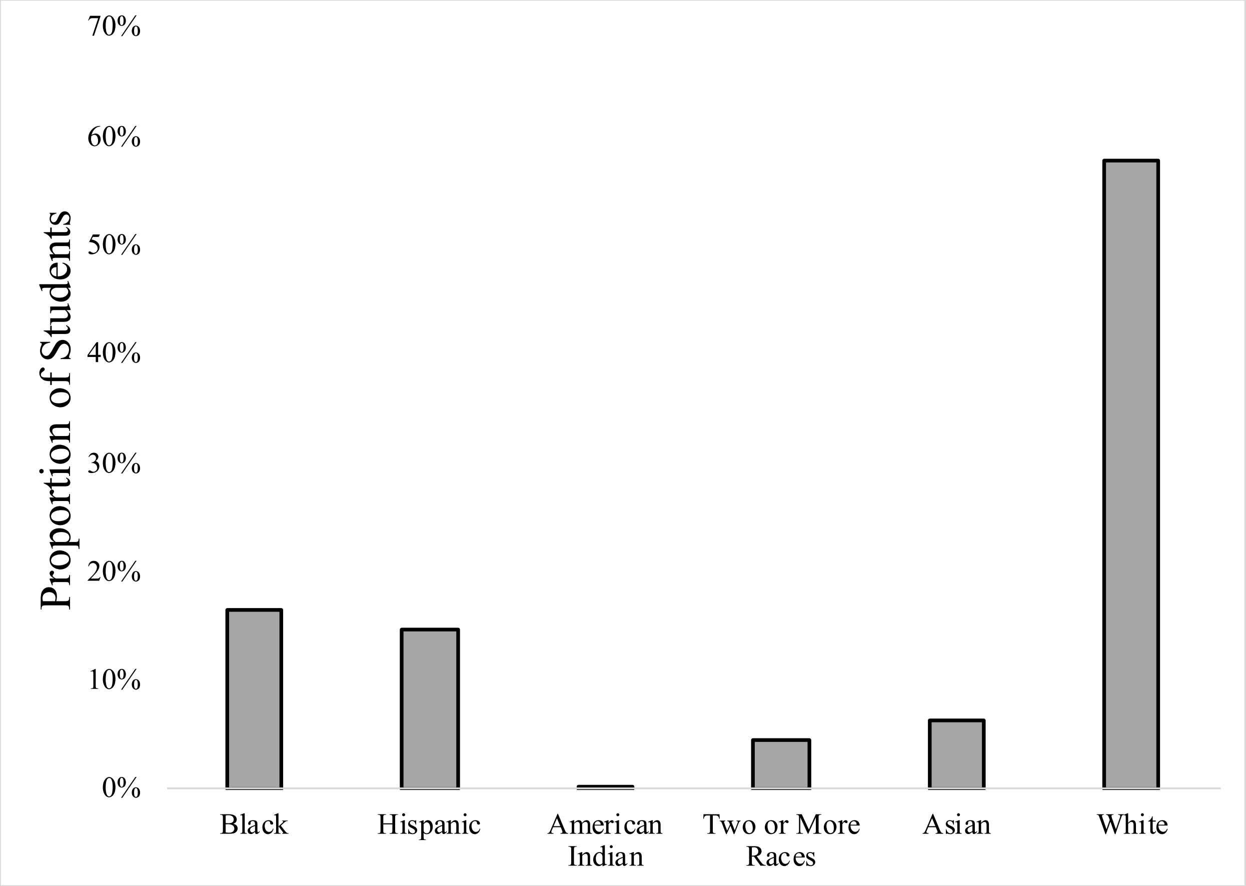

Note. Data are from the 5,822 students of teachers assigned to condition during the implementation period.

Figure S3. *Suspensions During the Year Prior to the Intervention Year as a Function of Race-Ethnicity in the Intervention Year Sample*

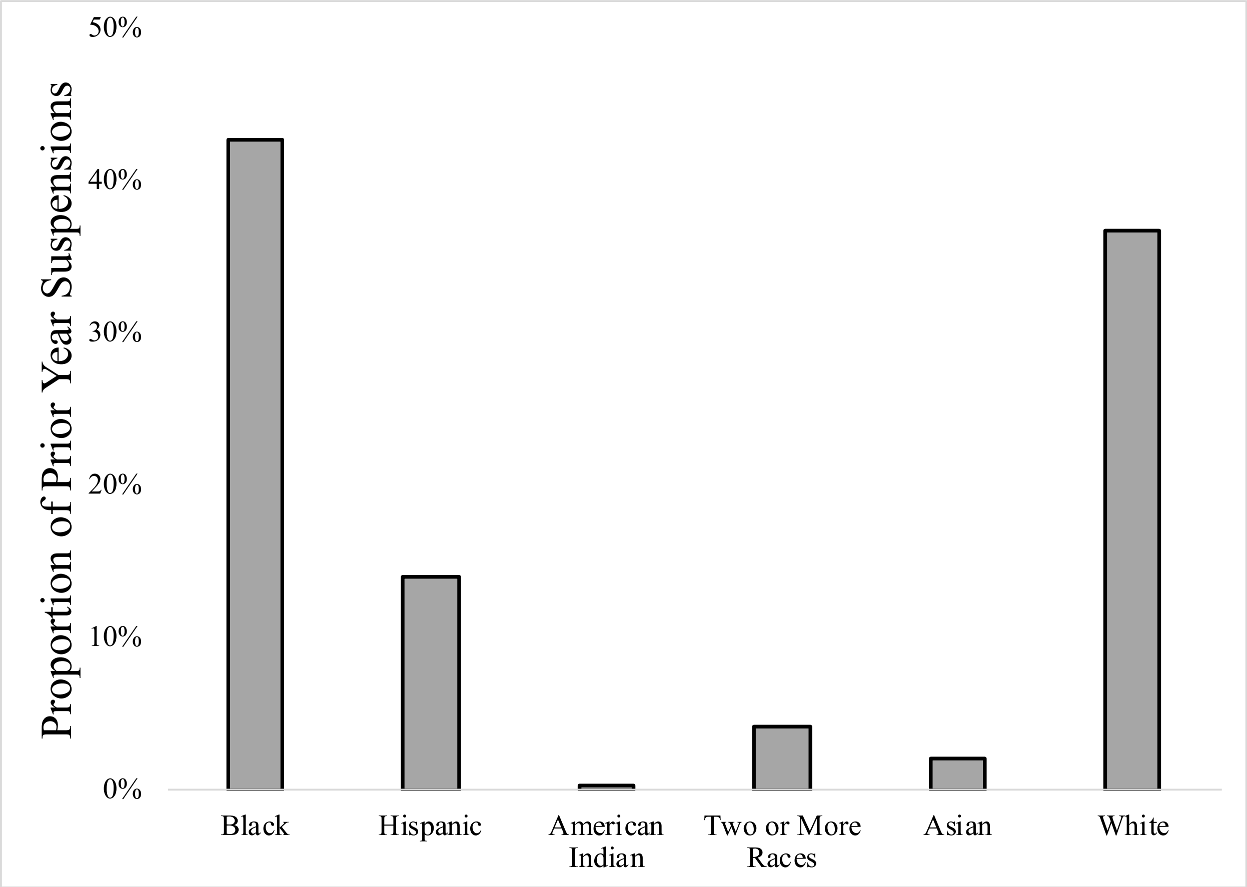

Note. Data are from the 870 students of teachers assigned to condition during the implementation period who had received a suspension the previous year.
